# A Strongly Unforgeable Certificateless Signature Scheme and Its Application in IoT Environments

**DOI:** 10.3390/s19122692

**Published:** 2019-06-14

**Authors:** Xiaodong Yang, Xizhen Pei, Guilan Chen, Ting Li, Meiding Wang, Caifen Wang

**Affiliations:** Computer Science and Engineering, Northwest Normal University, Lanzhou 730070, China; pxz0626@163.com (X.P.); chenglpaper@163.com (G.C.); lt1358382@163.com (T.L.); wmding95@163.com (M.W.); wangcfen@126.com (C.W.)

**Keywords:** certificateless signature, the Internet of Things, data integrity, data authenticity, strong unforgeability, provable security

## Abstract

With the widespread application of the Internet of Things (IoT), ensuring communication security for IoT devices is of considerable importance. Since IoT data are vulnerable to eavesdropping, tampering, forgery, and other attacks during an open network transmission, the integrity and authenticity of data are fundamental security requirements in the IoT. A certificateless signature (CLS) is a viable solution for providing data integrity, data authenticity, and identity identification in resource-constrained IoT devices. Therefore, designing a secure and efficient CLS scheme for IoT environments has become one of the main objectives of IoT security research. However, the existing CLS schemes rarely focus on strong unforgeability and replay attacks. Herein, we design a novel CLS scheme to protect the integrity and authenticity of IoT data. In addition to satisfying the strong unforgeability requirement, the proposed scheme also resists public key replacement attacks, malicious-but-passive key-generation-centre attacks, and replay attacks. Compared with other related CLS schemes without random oracles, our CLS scheme has a shorter private key, stronger security, and lower communication and computational costs.

## 1. Introduction

**Figure 1:**
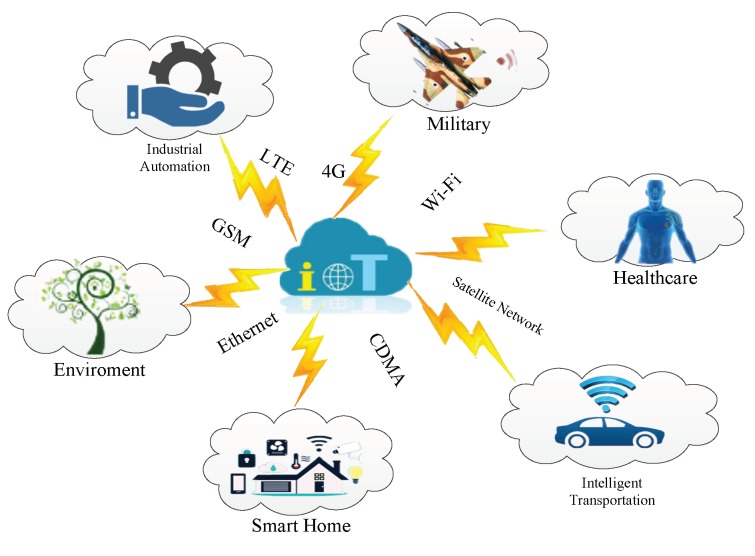
Internet of Things (IoT) applications.

The Internet of Things (IoT) is a self-establishing network of smart devices that are equipped with electronics, sensors, software, and actuators and that are connected via the Internet to generate, collect, and exchange data [1]. Since IoT devices connect objects in different environments to the Internet for information exchange and communication to realize intelligent identification, location, tracking, monitoring, management, and other functions, IoT devices have the ability to support a wide range of services. Consequently, the IoT builds a network that covers various things throughout the world via numerous IoT devices, and it enables various human-to-human, human-to-thing, thing-to-thing, and thing-to-thing interactions. Figure 1 shows a variety of IoT applications, including intelligent transportation, military target tracking, surveillance, public safety, smart home, industrial monitoring, smart city, medical equipment, and food traceability [2]. The application of the IoT involves all economic and social aspects of daily life and fundamentally changes the way in which humans interact with the world around them. Hence, the IoT is considered to be an information technology revolution and has become a growth point for the global economy [3].

Various IoT-enabled devices with embedded sensors collect and send IoT data to data centres over public networks; thus, the issues of security and privacy in IoT environments have become increasingly important [4,5]. Only authentic data can be stored in data centres, which requires the integrity and authenticity of the data transmitted by an IoT device to be checked before being stored. The signature-based cryptosystem is technology that provides the integrity, real source, unforgeability, and non-repudiation of the data. An IoT device signs the data using its private key during data transmission, and the data centre confirms the data authenticity and integrity by verifying the validity of the received signature. Therefore, a digital signature scheme can ensure data integrity and data authenticity in the IoT. However, the IoT differs from traditional networks. Most IoT devices have limited computational and processing capabilities, short communication ranges, and restricted storage and power resources. Conventional cryptosystems cannot run on resource-constrained IoT devices. The main reason is that conventional cryptosystems are classified into two categories: PKI-based and ID-based cryptosystems. Traditional PKI-based cryptosystems require certificates to authenticate users’ public keys, which results in a large amount of computational overhead and communication costs to manage and exchange certificates. The identity-based cryptosystem avoids the use of certificates, but there are security flaws in key escrow that make it unsuitable for large-scale network environments. Hence, designing an efficient, secure signature scheme is very important for IoT security.

In a signature scheme, the private key of the signer is used to sign the message, and the validity of the corresponding signature is verified by the signer’s public key. The signature’s validity not only ensures that the signer with the private key can apply a valid signature to the message but also ensures the authenticity and integrity of the message. The user’s public key is generally a random string; thus, authenticating the authenticity of the user’s public key is particularly crucial. In traditional public key infrastructure (PKI) settings, a certificate issued by a fully trusted authority associates the user’s public key with the user’s real identity. The authenticity of the user’s public key can be verified by the legality of the corresponding certificate. However, the storage, distribution, verification, and revocation of certificates in PKI are resource-intensive and computationally expensive tasks [6]. Hence, PKI is unsuitable for resource-constrained IoT environments.

Shamir [7] proposed identity-based cryptography (IBC) to solve the complex certificate management problems in PKI. IBC allows a key generation centre (KGC) to produce the user’s private key, but the corresponding user’s public key comes from their public identity information, such as an e-mail address or mobile phone number. However, KGC can replace the user to decrypt any ciphertext or to forge the signature of any message without being found, which results in the key escrow problem.

The concept of certificateless signature (CLS) was introduced by Al-Riyami and Paterson [8]. In a CLS scheme, the user’s private key consists of two parts: One is a partial private key generated by the KGC, and the other is a secret value calculated independently by the user. The CLS scheme solves the key escrow problem because the KGC is unable to obtain the user’s final private key. In addition, the user generates the corresponding public key based on its secret value, but it is not necessary to verify the authenticity of the public key by using the certificate. In practical applications, the user’s public key is sent to the recipient together with the signature or is obtained from a public directory in a proper manner.

Certificateless signatures have received considerable attention in recent years, and researchers have designed numerous CLS schemes [9,10,11,12]. Most existing CLS schemes [13,14,15] have been proven to be secure in the random oracle model [16], where a cryptographic hash function is modelled as an ideal random oracle. The random oracle paradigm helps construct efficient cryptographic schemes, but it has received substantial criticism. It has been shown that, when random oracles are instantiated with actual hash functions, the cryptographic scheme that proves to be secure using the random oracle model may be unsafe in reality [17]. To overcome this security flaw, Liu et al. [18] designed the first CLS scheme without random oracles. Later, several CLS schemes [19,20,21,22] in the standard model were proposed, but these schemes cannot resist public key replacement (PKR) attacks or malicious-but-passive KGC (MKGC) attacks. In addition, most existing CLS schemes [23,24,25] without random oracles are proven to be existentially unforgeable against adaptive chosen-message attacks. This security notion only ensures that an attacker cannot forge the signature of any new message; it does not guarantee that the attacker generates the valid signature for the signed message. However, some signature schemes are malleable [26]; thus, an attacker can generate multiple valid signatures of the same message by using the previous message–signature pair without the signer’s private key. In other words, these schemes do not satisfy strong unforgeability, which is a stronger security notion than existential unforgeability. Strong unforgeability is desirable in some applications [27,28,29] (such as electronic commerce, construction of certificateless signcryption schemes, and certificateless group signature schemes). If a CLS signature scheme satisfies existential unforgeability and can prevent an attacker from forging a valid signature of a previously signed message, then we say that the CLS scheme is strongly unforgeable. Strong unforgeability is an important property of the CLS scheme, but few CLS schemes [30] satisfy strong unforgeability in the standard model. Unfortunately, none of those strongly unforgeable CLS schemes considers replay attacks [31,32]. Note that the energy of the IoT device is one of the main factors that restricts improvements in network performance. However, replay attacks, which are considered to be one of the major attacks faced by IoT devices, can consume a large amount of node energy. Therefore, a CLS scheme that is applicable to IoT environments must consider replay attacks.

In this paper, motivated by the above concerns, we present a new CLS scheme for IoT environments that is more secure and efficient than the previous CLS schemes. As a potential signature-based authentication technology, our proposed scheme manifests a solution to the problems of data authenticity and data integrity in the IoT. The main contributions of this paper are the following.

- A novel CLS scheme without random oracles is constructed. Under the collision-resistant hash function (CRHF) and computational Diffie–Hellman (CDH) assumptions, the proposed CLS scheme is proven to be strongly unforgeable against adaptive chosen-message attacks in the standard model.
- In our CLS scheme, the user’s public key is not only bound to the user’s partial private key but also embedded into the signature of the message. This makes the proposed CLS scheme have a higher security trust level and be capable of resisting PKR attacks and MKGC attacks.
- The proposed CLS scheme resists replay attacks by verifying the freshness of the timestamp and the validity of the signature. To our best knowledge, our scheme is the first CLS scheme with a strong unforgeability in the standard model that can resist replay attacks.
- Compared to other CLS schemes in the standard model, our CLS scheme has higher security, a smaller key size, a shorter signature length, and lower computational overhead for signature generation and signature verification.
- Due to the aforementioned functionalities, our CLS scheme is able to be implemented and deployed in IoT environments where IoT devices have limited computing power, storage space, and communication bandwidth.

The remainder of this paper is organized as follows. We present the relevant CLS works in Section 2. Then, we introduce some preliminaries and security notions of the CLS scheme in Section 3. The proposed CLS scheme and its security proof are presented in Section 4 and Section 5. Section 6 gives the CLS system model for IoT environments and performance analysis. Section 7 concludes this paper.

## 2. Related Work

The first CLS scheme was proposed by Al-Riyami and Paterson [8]. Later, Huang et al. [33] noted that their CLS scheme [8] was unable to resist PKR attacks and proposed security notions for CLS schemes. Since then, researchers have constructed a large number of provably secure CLS schemes [9,10,11,12,13,14,15] in the random oracle model. Aiming to eliminate the security requirements of ideal random oracles, Liu et al. [18] constructed a CLS scheme without random oracles based on the identity-based signature scheme proposed by Paterson and Schuldt [34]. However, Xiong et al. [19] and Huang et al. [35] demonstrated that Liu et al.’s CLS scheme [18] was insecure against MKGC attacks. To enhance the security of Liu et al.’s CLS scheme [18], Xiong et al. [19] presented an improved scheme, but it was still vulnerable to MKGC attacks [36]. Furthermore, Xia et al. [37] showed that several CLS schemes [18,19,20] without random oracles were susceptible to PKR attacks.

Subsequently, Yu et al. [21] designed a CLS scheme and claimed that their scheme was secure in the standard model. However, Yuan et al. [23] and Pang et al. [27] independently demonstrated that Yu et al.’s CLS scheme [21] was insecure against PKR or MKGC attacks. As a countermeasure, Yuan et al. [23] designed an enhanced scheme, but it did not satisfy strong unforgeability. Based on the Boneh–Boyen signature [38] and Pointcheval–Sanders signature [39], Canard and Trinh [25] constructed a CLS scheme with a low computational cost. However, Canard and Trinh’s CLS scheme [25] was existentially unforgeable in the standard model. Subsequently, Huang et al. [22] constructed a CLS scheme with strong unforgeability in the standard model. Unfortunately, Yang et al. [30] demonstrated that Huang et al.’s CLS scheme [22] failed to achieve a strong unforgeability and was vulnerable to MKGC attacks. Furthermore, Yang et al. [30] presented a secure CLS scheme, but their scheme still has some drawbacks, including a longer private key size and a higher computational overhead than those of the previous schemes.

Digital signatures are widely used to ensure data authenticity and integrity. Yeh et al. [4] devised a CLS scheme for IoT environments. However, Jia et al. [40] demonstrated that Yeh et al.’s scheme [4] was insecure against PKR attacks and then proposed a new CLS scheme to overcome the flaws of Yeh et al.’s scheme [4]. Based on technologies such as RSA, DSA, and Merkle tree, Li et al. [41] proposed an IoT data communication framework to provide integrity and authenticity. Frädrich et al. [42] used redactable signature [43] to design another framework for the IoT environment to allow the redaction of parts from signed data and proved its security in the random oracle model. To achieve the security requirements in IoT, Challa et al. [44] presented a new signature-based authenticated key establishment scheme for the IoT environment. Based on Nyberg’s fast one-way accumulator [45], Yao et al. [46] designed a lightweight multicast authentication mechanism for small scale IoT applications. Yang et al. [47] proposed a certificateless aggregate signature scheme for vehicular ad hoc networks to reduce transmission bandwidth and verification overhead of signatures. To protect the identity privacy of IoT devices, Yang et al. [48] constructed a strong designated-verifier proxy re-signature (SDVPRS) scheme in the standard model and applied it to the IoT environment. Unfortunately, the existing data integrity and authenticity schemes in IoT have two drawbacks. (1) Some schemes [41,42,44,46,47,48] require heavy management and communication overheads of certificates to achieve authenticity authentication of the user’s public key. (2) Most of the schemes [4,40,41,42,44,46,47] are proved to be secure in the random oracle model. To fill thess gaps, Karati et al. [3] presented a secure CLS scheme for IoT environments in the standard model, but their scheme did not consider a strong unforgeability and replay attacks. To our best knowledge, designing an efficient CLS scheme that both satisfies strong unforgeability in the standard model and is resistant to PKR attacks, MKGC attacks, and replay attacks remains an open issue. Therefore, in this paper, we advance such a construction for IoT environments to ensure data integrity and data authenticity.

## 3. Preliminaries

Here, we briefly review some preliminary knowledge, including the definition of bilinear pairings, the complexity assumptions, and the security model of the CLS schemes.

### 3.1. Bilinear Paring

Assume that $G_{1}$ and $G_{2}$ are cyclic groups with the same order of prime *p* and that *g* is any generator of $G_{1}$. A bilinear pair $e:G_{1}\times G_{1}\to G_{2}$ is a map that satisfies the following conditions [23]:

- Bilinearity: $e\left(g^{a},g^{b}\right)=e{\left(g,g\right)}^{ab}$ for all $a,b\in Z_{p}$.
- Nondegeneracy: e(g,g)≠1.
- Computability: There is an algorithm that can efficiently calculate $e(g^{a},g^{b})$ for any $a,b\in Z_{p}$.

### 3.2. Complexity Assumptions

Given two elements $g,h\in G_{1}$, the discrete logarithm (DL) problem [30] is to find an integer $a\in Z_{p}$ such that $h=g^{a}$.

Let A denote an attacker with probabilistic polynomial time (PPT). The advantage ε of A to solve the DL problem in $G_{1}$ is defined as

$$Adv_{A}^{DL}=\mathrm{Pr}\left[A\left(g,h\right)=a:a\in Z_{p}\right]\geq \varepsilon .$$

**Definition** **1** (DL assumption)**.** *We say that the DL assumption holds in $G_{1}$ if there is no PPT attacker A to solve the DL problem with a non-negligible advantage ε.*

Given three elements, $g,g^{a},and\;g^{b}\in G_{1}$ for unknown, randomly chosen $a,b\in Z_{p}$, the computational Diffie–Hellman (CDH) problem [22] is to calculate $g^{ab}\in G_{1}$.

The advantage ε that any PPT adversary A can solve the CDH problem in $G_{1}$ is defined as

$$Adv_{A}^{CDH}=\mathrm{Pr}\left[A\left(g,g^{a},g^{b}\right)=g^{ab}:a,b\in Z_{p}\right]\geq \varepsilon .$$

**Definition** **2** (CDH assumption)**.** *The CDH assumption holds in $G_{1}$ if there is no PPT attacker A to solve the CDH problem with a non-negligible advantage ε.*

Suppose that $\left\{H_{k}\right\}$ represents a family of hash functions $H_{k}:{\left\{0,1\right\}}^{*}\to {\left\{0,1\right\}}^{n}$, where *n* is the length of the output value of $H_{k}$ and *k* is an index. Given the index *k*, the collision resistance of hash function (CRHF) [30] $H_{k}$ is to find $m_{0}\neq m_{1}$ such that $H_{k}\left(m_{0}\right)=H_{k}\left(m_{1}\right)$. The advantage ε of any PPT adversary A in breaking the collision resistance of $H_{k}$ is defined as

$$\begin{array}{cc} Adv_{A}^{CRHF}=\mathrm{Pr} & [A\left(k\right)=\left(m_{0},m_{1}\right):m_{0}\neq m_{1},H_{k}\left(m_{0}\right)=H_{k}\left(m_{1}\right)]\geq \varepsilon . \end{array}$$

**Definition** **3** (CRHF assumption)**.** *A hash family $\left\{H_{k}\right\}$ is collision resistant if the advantage ε of any PPT adversary A to break the collision resistance of hash function $H_{k}$ is negligible.*

### 3.3. Security Model of CLS

A CLS scheme consists of six algorithms, as follows:

- Setup: This algorithm takes as input a security parameter λ, and it outputs the master secret key msk and system parameters param.
- SetPubKey: This algorithm takes as input param and an identity ID, and it outputs a secret value $usk_{ID}$ and a public key $pk_{ID}$.
- PSKExtract: This algorithm takes as input param, ID, and $pk_{ID}$, and it returns a partial private key $psk_{ID}$ for identity ID.
- SetSecKey: Upon receiving param, $usk_{ID}$, and $psk_{ID}$, this algorithm outputs a private key $sk_{ID}$.
- Sign: This algorithm takes as input param, an identity ID’s private key $sk_{ID}$ and public key $pk_{ID}$, a timestamp *T*, and a message *m*, and it returns a signature σ on *m*.
- Verify: Upon receiving param, ID, $pk_{ID}$, *T*, *m*, and σ, this algorithm outputs 1 if σ is a valid signature of ID on *m* with respect to *T* and $pk_{ID}$, and it outputs 0 otherwise.

According to the security model for CLS presented in References [15,35], a CLS scheme’s security should consider two types of adversaries: type I and type II adversaries. A type I adversary is a PKR attacker who knows the secret value of the targeted entity and who can replace any entity’s public key with its own. A type I adversary models an outside attacker who is not capable of possessing the master secret key of the KGC. In contrast, a type II adversary models an honest-but-curious KGC attacker who holds the master secret key of the KGC and generates the partial private key of any entity. However, a type II adversary can neither perform the entity’s PKR nor obtain the secret value of the targeted entity. To meet more realistic security requirements, Au et al. [49] presented an enhanced security model in which a type II adversary is viewed as an MKGC attacker. In this case, a malicious KGC can access the master secret key of the KGC and may embed extra trapdoors in the system parameters and the master secret key during the initialization phase of the system. Hence, the type II adversary that we focus on is an MKGC attacker. Here, the security model for a strongly secure CLS scheme is formalized via the following games (denoted Games 1 and 2) between a challenger C and an adversary $A\in \{A_{1},A_{2}\}$.

**Game 1:** Executed between a challenger C and a type I adversary $A_{1}$.

- *Initialization*: C first runs the algorithm Setup to obtain the master secret key msk and system parameters param. C then runs the algorithm SetPubKey to output the secret value $usk^{*}$ and corresponding public key $pk^{*}$ of the targeted entity. Finally, C sends param and $\left(usk^{*},pk^{*}\right)$ to $A_{1}$ while keeping msk secret.
- *Queries*: $A_{1}$ can adaptively access the following oracles with C.
  – *Public Key Query*$O_{pk}\left(ID_{i}\right)$: Upon receiving an identity $ID_{i}$, C runs the algorithm SetPubKey to obtain a public key $pk_{i}$ and sends it to $A_{1}$.
  – *Public Key Replacement (PKR) Query*$O_{rep}\left(ID_{i},pk_{i}^{{}^{\prime }}\right)$: Upon receiving such a query, C finds and replaces the original public key $pk_{i}$ of identity $ID_{i}$ with a new public key $pk_{i}^{{}^{\prime }}$.
  – *Partial Private Key Query*$O_{psk}\left(ID_{i},pk_{i}\right)$: Upon receiving an identity $ID_{i}$ and a public key $pk_{i}$, C runs the algorithm PSKExtract to generate a partial private key $psk_{i}$ and sends it to $A_{1}$.
  – *Private Key Query*$O_{sk}\left(ID_{i}\right)$: When $A_{1}$ initiates a private key inquiry about an identity $ID_{i}$, C executes the algorithm SetSecKey to produce a private key $sk_{i}$ and sends it to $A_{1}$. Note that C returns the symbol ⊥ if $ID_{i}$ has already appeared in PKR queries.
  – *Signing Query*$O_{sign}\left(ID_{i},T,m\right)$: Upon receiving an identity $ID_{i}$, a timestamp *T*m and a message *m*, C first executes the algorithm SetSecKey to produce a private key $sk_{i}$ and then uses $sk_{i}$, *T*, and the identity $ID_{i}$’s matching public key $pk_{i}$ to execute the algorithm Sign to produce a signature $\sigma_{i}$ on *m*. Finally, C sends $\sigma_{i}$ to $A_{1}$.
- *Forgery*: $A_{1}$ eventually outputs a forged signature $\sigma^{*}$ on a message $m^{*}$ corresponding to an identity $ID^{*}$, a timestamp $T^{*}$, and the targeted public key $pk^{*}$. It is said that $A_{1}$ wins this game when the following conditions are fulfilled:
  (1) $\mathrm{Verify}(param,ID^{*},pk^{*},T^{*},m^{*},\sigma^{*})=1$.
  (2) $ID^{*}$ is not requested in $O_{psk}\left(ID_{i},pk_{i}\right)$ and $O_{sk}\left(ID_{i}\right)$.
  (3) $\left(ID^{*},T^{*},m^{*},\sigma^{*}\right)$ is not an output of the oracle $O_{sign}\left(ID_{i},T,m\right)$.

**Game 2:** Executed between a challenger C and a type II adversary $A_{2}$. To launch malicious attacks more easily, $A_{2}$ is allowed to set some trapdoors during the initialization phase of the game.

- *Initialization*: C invokes $A_{2}$ to produce the master secret key msk and system parameters param. Then, C runs the algorithm SetPubKey to produce the secret value $usk^{*}$ and the corresponding public key $pk^{*}$ of the targeted entity. Finally, C sends $pk^{*}$ to $A_{2}$ while keeping $usk^{*}$ secret.
- *Queries*: $A_{2}$ can adaptively access the oracles $O_{pk}\left(ID_{i}\right)$, $O_{sk}\left(ID_{i}\right)$, and $O_{sign}\left(ID_{i},T,m\right)$, which are defined in Game 1, and C responds in the same way as it does in Game 1.
- *Forgery*: $A_{2}$ eventually outputs a forged signature $\sigma^{*}$ on a message $m^{*}$ corresponding to an identity $ID^{*}$, a timestamp $T^{*}$, and the targeted public key $pk^{*}$. It is said that $A_{2}$ wins this game when the following conditions are fulfilled:
  (1) $\mathrm{Verify}(param,ID^{*},pk^{*},T^{*},m^{*},\sigma^{*})=1$.
  (2) $ID^{*}$ is not requested in $O_{sk}\left(ID_{i}\right)$.
  (3) $\left(ID^{*},T^{*},m^{*},\sigma^{*}\right)$ is not an output of the oracle $O_{sign}\left(ID_{i},T,m\right)$.

$Adv_{A_{i}}^{SUF}=\mathrm{Pr}\left[A_{i}\;succeeds\right]$ denotes the advantage that $A_{i}$ wins the above games, where i∈{1,2}.

**Definition** **4.** *A CLS scheme is said to be strongly unforgeable against adaptive chosen message attacks if the advantages $Adv_{A_{1}}^{SUF}$ and $Adv_{A_{2}}^{SUF}$ are negligible for all PPT type I adversaries $A_{1}$ and type II adversaries $A_{2}$.*

## 4. Proposed CLS Scheme

Based on Waters’ scheme [26] and its variants [28,34], we propose an undeniable and strongly unforgeable CLS scheme in the standard model. Our CLS scheme is described as follows.

- Setup: Upon giving the security parameter λ as input, the KGC produces the master secret key and system parameters by performing the following steps.
  (1) Select $G_{1}$ and $G_{2}$ as two cyclic groups with prime order *p*, a generator *g* of $G_{1}$, and a bilinear pairing $e:G_{1}\times G_{1}\to G_{2}$.
  (2) Select two random values $\alpha ,\beta \in Z_{p}^{*}$ and compute $g_{1}=g^{\alpha }$ and $g_{2}=g^{\beta }$.
  (3) Select two random elements $u_{0},v_{0}\in G_{1}$ and two vectors $\vec{u}=\left(u_{i}\right)$ and $\vec{v}=\left(v_{j}\right)$ of lengths $n_{u}$ and $n_{m}$, respectively, where $u_{i},v_{j}\in G_{1}$ for $i=1,\ldots ,n_{u}$ and $j=1,\ldots ,n_{m}$.
  (4) Select three collision-resistant hash functions $H_{1}:{\left\{0,1\right\}}^{*}\to {\left\{0,1\right\}}^{n_{u}}$, $H_{2}:{\left\{0,1\right\}}^{*}\to {\left\{0,1\right\}}^{n_{m}}$, and $H_{3}:{\left\{0,1\right\}}^{*}\to Z_{p}^{*}$.
  (5) Secretly keep the master key $msk=g^{\alpha \beta }$ and publicly broadcast the system parameters $param=\{G_{1},G_{2},p,g$, $e,g_{1},g_{2},u_{0},v_{0},\vec{u},\vec{v},H_{1},H_{2},H_{3}\}$.
- SetPubKey: An entity with identity ID randomly selects $\theta_{1},\theta_{2},\theta_{3}\in Z_{p}^{*}$ and computes
  $$pk_{ID,1}=g^{\theta_{1}},\;pk_{ID,2}=g^{\theta_{2}}\;\mathrm{and}\;pk_{ID,3}=g^{\theta_{3}}.$$
  Then, the entity computes $usk_{ID}=g^{\theta_{1}\theta_{2}}$ as its secret value and sets its public key $pk_{ID}=\left(pk_{ID,1},pk_{ID,2},pk_{ID,3}\right)$.
- PSKExtract: Given an identity ID and a public key $pk_{ID}$ of an entity, the KGC first computes a vector $\vec{Q}=H_{1}\left(ID,pk_{ID}\right)=\left(Q_{1},\ldots ,Q_{n_{u}}\right)\in {\left\{0,1\right\}}^{n_{u}}$ and $U_{ID}=u_{0}\prod_{i=1}^{n_{u}}u_{i}^{Q_{i}}$. Then, the KGC selects $s\in Z_{p}^{*}$ at random and computes
  $$psk_{ID,1}=g^{\alpha \beta }{\left(U_{ID}\right)}^{s}\;\mathrm{and}\;psk_{ID,2}=g^{s}.$$
  Finally, the KGC sends the partial private key $psk_{ID}=\left(psk_{ID,1},psk_{ID,2}\right)$ to the entity via a secure channel.
  After receiving $psk_{ID}=\left(psk_{ID,1},psk_{ID,2}\right)$ from the KGC, the entity can check the correctness of $psk_{ID}$ by verifying
  $$e\left(psk_{ID,1},g\right)=e\left(g_{2},g_{1}\right)e\left(U_{ID},psk_{ID,2}\right).$$
  If this equation holds, then the entity accepts $psk_{ID}$ as a valid partial private key.
- SetSecKey: The entity with identity ID selects a random value $r\in Z_{p}^{*}$ and computes a vector $\vec{Q}=H_{1}\left(ID,pk_{ID}\right)=\left(Q_{1},\ldots ,Q_{n_{u}}\right)\in {\left\{0,1\right\}}^{n_{u}}$ and $U_{ID}=u_{0}\prod_{i=1}^{n_{u}}u_{i}^{Q_{i}}$, where $pk_{ID}$ is ID’s public key. Then, the entity uses its secret value $usk_{ID}$ and partial private key $psk_{ID}=\left(psk_{ID,1},psk_{ID,2}\right)$ to compute its private key
  $$\begin{array}{cc} sk_{ID} & =(sk_{ID,1},sk_{ID,2})\\ & =(psk_{ID,1}\cdot usk_{ID}\cdot {\left(U_{ID}\right)}^{r},psk_{ID,2}\cdot g^{r})\\ & =(g^{\alpha \beta }{\left(U_{ID}\right)}^{s}\cdot g^{\theta_{1}\theta_{2}}\cdot {\left(U_{ID}\right)}^{r},g^{s}\cdot g^{r})\\ & =(g^{\alpha \beta }g^{\theta_{1}\theta_{2}}{\left(U_{ID}\right)}^{s+r},g^{s+r}). \end{array}$$
- Sign: The signer with identity ID generates a signature of a message *m* by performing the following steps.
  (1) Select a random value $r_{m}\in Z_{p}^{*}$ and compute $\sigma_{3}=g^{r_{m}}$.
  (2) Choose the current timestamp *T* and compute a vector $\vec{M}=H_{2}\left(m,T\right)=\left(M_{1},\ldots ,M_{n_{m}}\right)\in {\left\{0,1\right\}}^{n_{m}}$ and $V_{m}=v_{0}\prod_{j=1}^{n_{m}}v_{j}^{M_{j}}$.
  (3) Compute
    $$\begin{array}{cc} h & =H_{3}\left(m,T,ID,pk_{ID},sk_{ID,2},\sigma_{3},param\right),\\ \sigma_{1} & =sk_{ID,1}\cdot {\left({\left(pk_{ID,3}\right)}^{h}V_{m}\right)}^{r_{m}},\\ \sigma_{2} & =sk_{ID,2}, \end{array}$$
    where $sk_{ID}=\left(sk_{ID,1},sk_{ID,2}\right)$ and $pk_{ID}=$ $\left(pk_{ID,1},pk_{ID,2},pk_{ID,3}\right)$ are the private and public keys of identity ID, respectively.
  (4) Output $\sigma =(\sigma_{1},\sigma_{2},\sigma_{3})$ as a signature of *m*.
- Verify: Given the signer’s identity ID and public key $pk_{ID}=$ $\left(pk_{ID,1},pk_{ID,2},pk_{ID,3}\right)$, timestamp *T*, and a signature $\sigma =(\sigma_{1},\sigma_{2},\sigma_{3})$ of message *m*, the verifier first chooses the current time $T^{{}^{\prime }}$. Then, the verifier verifies the legality of σ as follows.
  (1) If $T^{{}^{\prime }}-T>\delta$, where δ is a threshold value, the verifier refuses to verify the validity of σ and exits.
  (2) If $T^{{}^{\prime }}-T\leq \delta$, the verifier computes $\vec{Q}=H_{1}\left(ID,pk_{ID}\right)$, $U_{ID}$, $\vec{M}=H_{2}\left(m,T\right)$, $V_{m}$ and
    $$h=H_{3}\left(m,T,ID,pk_{ID},sk_{ID,2},\sigma_{3},param\right).$$
    Then, the verifier checks
    $$\begin{array}{cc} e(\sigma_{1},g)= & e\left(g_{2},g_{1}\right)\cdot e\left(pk_{ID,1},pk_{ID,2}\right)\cdot e\left(U_{ID},\sigma_{2}\right)\cdot e\left({\left(pk_{ID,3}\right)}^{h}V_{m},\sigma_{3}\right). \end{array}$$
    If this equation holds, the verifier accepts σ and outputs 1; otherwise, the verifier rejects σ and outputs 0.

*Correctness:* The correctness of a signature $\sigma =(\sigma_{1},\sigma_{2},\sigma_{3})$ on a message *m* is presented as follows:

$$\begin{array}{cc} e(\sigma_{1},g)= & e(sk_{ID,1}\cdot {\left({\left(pk_{ID,3}\right)}^{h}V_{m}\right)}^{r_{m}},g)\\ = & e(g^{\alpha \beta }g^{\theta_{1}\theta_{2}}{\left(U_{ID}\right)}^{s+r}\cdot {\left({\left(pk_{ID,3}\right)}^{h}V_{m}\right)}^{r_{m}},g)\\ = & e\left(g^{\alpha \beta },g\right)\cdot e\left(g^{\theta_{1}\theta_{2}},g\right)\cdot e\left({\left(U_{ID}\right)}^{s+r},g\right)\cdot e\left({\left({\left(pk_{ID,3}\right)}^{h}V_{m}\right)}^{r_{m}},g\right)\\ = & e\left(g^{\alpha },g^{\beta }\right)\cdot e\left(g^{\theta_{1}},g^{\theta_{2}}\right)\cdot e\left(U_{ID},g^{s+r}\right)\cdot e\left({\left(pk_{ID,3}\right)}^{h}V_{m},g^{r_{m}}\right)\\ = & e\left(g_{2},g_{1}\right)\cdot e\left(pk_{ID,1},pk_{ID,2}\right)\cdot e\left(U_{ID},\sigma_{2}\right)\cdot e\left({\left(pk_{ID,3}\right)}^{h}V_{m},\sigma_{3}\right). \end{array}$$

## 5. Security Proof

In our CLS scheme, the algorithm SetSecKey randomizes the entity’s secret value $usk_{ID}$ and partial private key $psk_{ID}=\left(psk_{ID,1},psk_{ID,2}\right)$ to generate the final private key $sk_{ID}=\left(sk_{ID,1},sk_{ID,2}\right)=\left(psk_{ID,1}\cdot usk_{ID}\cdot {\left(U_{ID}\right)}^{r},psk_{ID,2}\cdot g^{r}\right)$. Hence, it is not feasible for a malicious KGC to produce a valid signature without the secret value $usk_{ID}$. Additionally, the KGC cannot derive the entity’s private key $sk_{ID}$ from the master secret key msk and the entity’s partial private key $psk_{ID}$.

To prevent PKR and MKGC attacks, a part $pk_{ID,3}$ of the entity’s public key is embedded in the signature σ. Only each entity can produce its legal public key $pk_{ID}=\left(pk_{ID,1},pk_{ID,2},pk_{ID,3}\right)=\left(g^{\theta_{1}},g^{\theta_{2}},g^{\theta_{3}}\right)$; thus, the malicious KGC can neither set the entity’s public key at will nor derive the secret value $usk_{ID}$ from the signature. Furthermore, it is impossible to obtain the value ${\left(pk_{ID,3}\right)}^{r_{m}}$ directly from the entity’s public key $pk_{ID}$ and the public value $\sigma_{3}=g^{r_{m}}$ of the signature unless the adversary can solve the CDH problem.

The algorithm PSKExtract binds each entity’s public key $pk_{ID}$, identity ID, and partial private key $psk_{ID}$, which can enhance the trust level of the proposed CLS scheme. If the KGC attempts to replace the entity’s public key $pk_{ID}$, then the entity’s identity ID and the new public key must be re-bound to compute a new partial private key, which results in the entity’s identity ID corresponding to two public keys and two partial private keys. Therefore, our CLS scheme can easily determine whether the KGC replaced the entity’s public key.

In addition, $H_{3}$ is a collision-resistant hash function. The hash value *h* combines the message *m*, the identity ID, public key $pk_{ID}$, two values $\sigma_{2}$, and $\sigma_{3}$ in the signature, a timestamp *T*, and system parameters param as $h=H_{3}\left(m,T,ID,pk_{ID},sk_{ID,2},\sigma_{3},param\right)$. Hence, an attacker cannot forge a new valid signature from an existing signature on a message; that is, an adversary cannot generate a valid signature on any previously signed/new message in our CLS scheme.

In the following, we introduce two theorems to demonstrate that our CLS scheme satisfies a strong unforgeability against PKR and MKGC attacks in the standard model. Reduction technology is used to prove the strong unforgeability of the proposed scheme; specifically, if an attacker breaks the security of the scheme, a solver then uses the attacker’s ability to solve the underlying hard problem related to the scheme. However, this problem is intractable in reality; thus, such an attacker does not exist. Furthermore, we prove that the proposed CLS scheme can resist replay attacks.

**Theorem** **1.** *In the standard model, our CLS scheme is strongly unforgeable against PKR attacks. Specifically, there is a type I adversary $A_{1}$ that breaks the security of the proposed CLS scheme with advantage $\varepsilon_{1}$ after making at most $q_{pk}$ public key queries, $q_{psk}$ partial private key queries, $q_{rep}$ PKR queries, $q_{sk}$ private key queries, and $q_{s}$ signing queries. Then, an algorithm C can use the forgery of $A_{1}$ to solve the CDH problem with advantage $\varepsilon_{1}^{{}^{\prime }}$.*

**Proof.**  C is given a random instance $\left(g,g^{a},g^{b}\right)\in G_{1}^{3}$ of the CDH problem, and C’s goal is to output $g^{ab}$ with the help of $A_{1}$. The algorithm C simulates the challenger in Game 1 and responds to $A_{1}$’s queries as follows.

- *Initialization*: C first sets $l_{u}=2\left(q_{psk}+q_{sk}+q_{s}\right)$ and $l_{m}=2q_{s}$ such that $l_{u}\left(n_{u}+1\right)<p$ and $l_{m}\left(n_{m}+1\right)<p$. Then, C simulates the algorithm Setup by performing the following steps:
  (1) Randomly select $k_{u}$$\left(0\leq k_{u}\leq n_{u}\right)$ and $k_{m}$$\left(0\leq k_{m}\leq n_{m}\right)$.
  (2) Randomly select $x_{0},x_{1},\ldots ,x_{n_{u}}\in Z_{l_{u}}$, $y_{0},y_{1}$, $\ldots ,y_{n_{u}}\in Z_{p}$, $c_{0},c_{1},\ldots ,c_{n_{m}}\in Z_{l_{m}}$, and $d_{0},d_{1},\ldots$, $d_{n_{m}}\in \;Z_{p}$.
  (3) Select three hash functions $H_{1}:{\left\{0,1\right\}}^{*}\to {\left\{0,1\right\}}^{n_{u}}$, $H_{2}:{\left\{0,1\right\}}^{*}\to {\left\{0,1\right\}}^{n_{m}}$, and $H_{3}:{\left\{0,1\right\}}^{*}\to Z_{p}^{*}$. Note that the adopted hash functions are not considered to be random oracles in the following proof.
  (4) Set $g_{1}=g^{a}$ and $g_{2}=g^{b}$, where $g^{a}$ and $g^{b}$ are from the input of the instance of the CDH problem. Note that the master secret key is implicitly set to $msk=g^{ab}$.
  (5) Assign $u_{0}=g_{2}^{-l_{u}k_{u}+x_{0}}g^{y_{0}}$, $u_{i}=g_{2}^{x_{i}}g^{y_{i}}$ for $i=1,\ldots ,n_{u}$, $v_{0}=g_{2}^{-l_{m}k_{m}+c_{0}}g^{d_{0}}$, and $v_{j}=g_{2}^{c_{j}}g^{d_{j}}$ for $j=1,\ldots ,n_{m}$, and set $\vec{u}=\left(u_{1},\ldots ,u_{n_{u}}\right)$ and $\vec{v}=\left(v_{1},\ldots ,v_{n_{m}}\right)$.
  (6) Select three random integers $\theta_{1}^{*},\theta_{2}^{*},\theta_{3}^{*}\in Z_{p}^{*}$ and compute $pk_{1}^{*}=g^{\theta_{1}^{*}}$, $pk_{2}^{*}=g^{\theta_{2}^{*}}$, and $pk_{3}^{*}=g^{\theta_{3}^{*}}$. Next, set the secret value of the targeted entity to $usk^{*}=g^{\theta_{1}^{*}\theta_{2}^{*}}$ and the corresponding public key to $pk^{*}=\left(pk_{1}^{*},pk_{2}^{*},pk_{3}^{*}\right)$.
  (7) Send system parameters $param=\{G_{1},G_{2},p,g$, $e,g_{1},g_{2},u_{0},v_{0},\vec{u},\vec{v},H_{1},H_{2},H_{3}\}$ and the targeted entity’s secret value/public key pair $\left(usk^{*},pk^{*}\right)$ to $A_{1}$.
  From the perspective of $A_{1}$, the distribution of the system parameters produced by C is identical to the real construction.
  In our CLS scheme, we have $\vec{Q}=H_{1}\left(ID,pk_{ID}\right)=\left(Q_{1},\ldots ,Q_{n_{u}}\right)\in {\left\{0,1\right\}}^{n_{u}}$ for an identity ID and a public key $pk_{ID}$, and we have $\vec{M}=H_{2}\left(m,T\right)=(M_{1}$, $\ldots ,M_{n_{m}})\in {\left\{0,1\right\}}^{n_{m}}$ for a message *m* and a timestamp *T*. Aiming to simplify the analysis, we define the following four functions:
  $$\begin{array}{cc} F(ID) & =-l_{u}k_{u}+x_{0}+\sum_{i=1}^{n_{u}}x_{i}Q_{i},J\left(ID\right)=y_{0}+\sum_{i=1}^{n_{u}}y_{i}Q_{i},\\ K(m) & =-l_{m}k_{m}+c_{0}+\sum_{j=1}^{n_{m}}c_{j}M_{j},L\left(m\right)=d_{0}+\sum_{j=1}^{n_{m}}d_{j}M_{j}. \end{array}$$
  Hence, we have the following equations:
  $$\begin{array}{cc} U_{ID} & =u_{0}\prod_{i=1}^{n_{u}}u_{i}^{Q_{i}}=g_{2}^{F(ID)}g^{J(ID)},\\ V_{m} & =v_{0}\prod_{j=1}^{n_{m}}v_{j}^{M_{j}}=g_{2}^{K(m)}g^{L(m)}. \end{array}$$
- *Queries*: C maintains a list $L=\{(ID_{i},\theta_{i,1},\theta_{i,2},\theta_{i,3}$, $usk_{i},pk_{i},psk_{i},sk_{i})\}$, which is initially empty. C constructs the following oracles to answer a series of $A_{1}$’s queries.
  – *Public Key Query*$O_{pk}\left(ID_{i}\right)$: When $A_{1}$ initiates such an inquiry for an identity $ID_{i}$, C looks up the corresponding entry in the list L. If $ID_{i}$ is found in L, C returns $pk_{i}$ to $A_{1}$. Otherwise, C randomly selects $\theta_{i,1},\theta_{i,2},\theta_{i,3}\in Z_{p}^{*}$ and computes the secret value $usk_{i}=g^{\theta_{i,1}\theta_{i,2}}$ and the public key $pk_{i}=\left(pk_{i,1},pk_{i,2},pk_{i,3}\right)=\left(g^{\theta_{i,1}},g^{\theta_{i,2}},g^{\theta_{i,3}}\right)$. Then, C stores $\left\{\left(ID_{i},\theta_{i,1},\theta_{i,2},\theta_{i,3},usk_{i},pk_{i}\right)\right\}$ in L and sends $pk_{i}$ to $A_{1}$.
  – *Public Key Replacement Query*$O_{rep}\left(ID_{i},pk_{i}^{{}^{\prime }}\right)$: If there is an entry for the identity $ID_{i}$ in the list L, C replaces the original public key $pk_{i}$ of $ID_{i}$ with a new public key $pk_{i}^{{}^{\prime }}$. Otherwise, C directly sets $pk_{i}^{{}^{\prime }}$ as the public key of $ID_{i}$.
  – *Partial Private Key Query*$O_{psk}\left(ID_{i},pk_{i}\right)$: When $A_{1}$ requests a partial private key of an identity $ID_{i}$ and a public key $pk_{i}$, C returns $psk_{i}$ to $A_{1}$ if there is an entry for $ID_{i}$ and $pk_{i}$ in the list L. Otherwise, C computes $F(ID_{i})$ and $J(ID_{i})$.
    (1) If $F\left(ID_{i}\right)\neq 0\;\mathrm{mod}\;l_{u}$, C randomly selects $s_{i}\in Z_{p}^{*}$ and calculates a partial private key
      $$\begin{array}{cc} psk_{i} & =(psk_{i,1},psk_{i,2})\\ & =(g_{1}^{\frac{-J(ID_{i})}{F(ID_{i})}}{\left(U_{ID_{i}}\right)}^{s_{i}},g_{1}^{\frac{-1}{F(ID_{i})}}g^{s_{i}}), \end{array}$$
      where $U_{ID_{i}}=u_{0}\prod_{k=1}^{n_{u}}u_{k}^{Q_{i,k}}$ and ${\vec{Q}}_{i}=H_{1}(ID_{i}$, $pk_{i})=\left(Q_{i,1},\ldots ,Q_{i,n_{u}}\right)\in {\left\{0,1\right\}}^{n_{u}}$. Then, C stores the partial private key of the corresponding entry in L and sends $psk_{i}$ to $A_{1}$.
    (2) If $F\left(ID_{i}\right)=0\;\mathrm{mod}\;l_{u}$, C terminates the simulation.
    Note that the partial private key $psk_{i}=\left(psk_{i,1},psk_{i,2}\right)$ generated by C is legal.
    $$\begin{array}{cc} psk_{i,1} & =g_{1}^{\frac{-J(ID_{i})}{F(ID_{i})}}{\left(U_{ID_{i}}\right)}^{s_{i}}\\ & =g_{2}^{a}{\left(g_{2}^{F(ID_{i})}g^{J(ID_{i})}\right)}^{\frac{-a}{F(ID_{i})}}{\left(U_{ID_{i}}\right)}^{s_{i}}\\ & =g_{2}^{a}{\left(U_{ID_{i}}\right)}^{s_{i}-\frac{a}{F(ID_{i})}}\\ & =g^{ab}{\left(U_{ID_{i}}\right)}^{s_{i}-\frac{a}{F(ID_{i})}},\\ psk_{i,2} & =g_{1}^{\frac{-1}{F(ID_{i})}}g^{s_{i}}=g^{s_{i}-\frac{a}{F(ID_{i})}}. \end{array}$$
    Then, we have
    $$e\left(psk_{i,1},g\right)=e\left(g_{2},g_{1}\right)e\left(U_{ID_{i}},psk_{i,2}\right).$$
    Hence, from $A_{1}$’s perspective, the partial private key $psk_{i}$ simulated by C is computationally indistinguishable from that computed by the real KGC.
  – *Private Key Query*$O_{sk}\left(ID_{i}\right)$: When $A_{1}$ requests the private key of an identity $ID_{i}$, C checks for an entry of $ID_{i}$ in L. If it exists, C returns $sk_{i}$ to $A_{1}$; otherwise, C computes $F(ID_{i})$ and $J(ID_{i})$. If $F\left(ID_{i}\right)=0\;\mathrm{mod}\;l_{u}$, C terminates; otherwise, C initiates a public key query about $ID_{i}$ to acquire a secret value $usk_{i}$ and a public key $pk_{i}$ and then initiates a partial private key query with $\left(ID_{i},pk_{i}\right)$ to acquire a partial private key $psk_{i}$. Next, C executes the algorithm SetSecKey to create a private key $sk_{i}$, stores $sk_{i}$ of the corresponding entry in L and sends $sk_{i}$ to $A_{1}$.
  – *Signing Query*$O_{sign}\left(ID_{i},T,m\right)$: Upon receiving an identity $ID_{i}$, a timestamp *T*, and a message *m*, C issues a query $O_{pk}\left(ID_{i}\right)$ to acquire a public key $pk_{i}=\left(pk_{i,1},pk_{i,2},pk_{i,3}\right)$ and the triplet $\left(\theta_{i,1},\theta_{i,2},\theta_{i,3}\right)$. Then, C proceeds as follows.
    (1) If $F\left(ID_{i}\right)\neq 0\;\mathrm{mod}\;l_{u}$, C first makes a query $O_{sk}\left(ID_{i}\right)$ to acquire a private key $sk_{i}$ and then runs the algorithm Sign to generate a signature $\sigma_{i}$ of *m*. Finally, C sends $\sigma_{i}$ to $A_{1}$.
    (2) If $F\left(ID_{i}\right)=0\;\mathrm{mod}\;l_{u}$, C computes K(m). If $K\left(m\right)=0\;\mathrm{mod}\;l_{m}$, C terminates; otherwise, C randomly selects $r_{i},r_{m}\in Z_{p}^{*}$ and computes ${\vec{Q}}_{i}=H_{1}\left(ID_{i},pk_{i}\right)$, $U_{ID_{i}}$, $\vec{M}=H_{2}\left(m,T\right)$, and $V_{m}$. Furthermore, C computes
      $$\begin{array}{cc} \sigma_{i,2}= & g^{r_{i}},\\ \sigma_{i,3}= & g_{1}^{\frac{-1}{K(m)}}g^{r_{m}},\\ h_{i}= & H_{3}\left(m,T,ID_{i},pk_{i},\sigma_{i,2},\sigma_{i,3},param\right),\\ \sigma_{i,1}= & {\left(U_{ID_{i}}\right)}^{r_{i}}g_{1}^{\frac{-L\left(m\right)-h_{i}\theta_{i,3}}{K(m)}}{\left({\left(pk_{i,3}\right)}^{h_{i}}V_{m}\right)}^{r_{m}}g^{\theta_{i,1}\theta_{i,2}}. \end{array}$$
      Finally, C sends $\sigma_{i}=\left(\sigma_{i,1},\sigma_{i,2},\sigma_{i,3}\right)$ to $A_{1}$.
  For ${\tilde{r}}_{m}=r_{m}-\frac{a}{K(m)}$, we have
  $$\begin{array}{cc} \sigma_{i,1}= & {\left(U_{ID_{i}}\right)}^{r_{i}}g_{1}^{\frac{-L\left(m\right)-h_{i}\theta_{i,3}}{K(m)}}{\left({\left(pk_{i,3}\right)}^{h_{i}}V_{m}\right)}^{r_{m}}g^{\theta_{i,1}\theta_{i,2}}\\ = & {\left(U_{ID_{i}}\right)}^{r_{i}}g^{ab}{\left(g_{2}^{K(m)}g^{L(m)}g^{h_{i}\theta_{i,3}}\right)}^{\frac{-a}{K(m)}}{\left({\left(pk_{i,3}\right)}^{h_{i}}V_{m}\right)}^{r_{m}}g^{\theta_{i,1}\theta_{i,2}}\\ = & g^{ab}g^{\theta_{i,1}\theta_{i,2}}{\left(U_{ID_{i}}\right)}^{r_{i}}{\left({\left(pk_{i,3}\right)}^{h_{i}}V_{m}\right)}^{r_{m}-\frac{a}{K(m)}}\\ = & g^{ab}g^{\theta_{i,1}\theta_{i,2}}{\left(U_{ID_{i}}\right)}^{r_{i}}{\left({\left(pk_{i,3}\right)}^{h_{i}}V_{m}\right)}^{{\tilde{r}}_{m}},\\ \sigma_{i,2}= & g^{r_{i}},\\ \sigma_{i,3}= & g_{1}^{\frac{-1}{K(m)}}g^{r_{m}}=g^{r_{m}-\frac{a}{K(m)}}=g^{{\tilde{r}}_{m}}. \end{array}$$
  Clearly, the signature $\sigma_{i}=\left(\sigma_{i,1},\sigma_{i,2},\sigma_{i,3}\right)$ generated by C is legal because $\sigma_{i}$ satisfies the following verification equation:
  $$\begin{array}{cc} e(\sigma_{i,1},g)= & e(g^{ab}g^{\theta_{i,1}\theta_{i,2}}{\left(U_{ID_{i}}\right)}^{r_{i}}{\left({\left(pk_{i,3}\right)}^{h_{i}}V_{m}\right)}^{{\tilde{r}}_{m}},g)\\ = & e\left(g^{a},g^{b}\right)e\left(g^{\theta_{i,1}},g^{\theta_{i,2}}\right)e\left(U_{ID_{i}},g^{r_{i}}\right)e\left({\left(pk_{i,3}\right)}^{h_{i}}V_{m},g^{{\tilde{r}}_{m}}\right)\\ = & e\left(g_{2},g_{1}\right)\cdot e\left(pk_{i,1},pk_{i,2}\right)\cdot e\left(U_{ID_{i}},\sigma_{i,2}\right)e\left({\left(pk_{i,3}\right)}^{h_{i}}V_{m},\sigma_{i,3}\right). \end{array}$$
  From $A_{1}$’s perspective, the signatures simulated by C are computationally indistinguishable from those produced by the real signer.
- *Forgery*: $A_{1}$ eventually outputs a signature $\sigma^{*}=\left(\sigma_{1}^{*},\sigma_{2}^{*},\sigma_{3}^{*}\right)$ on a message $m^{*}$ corresponding to an identity $ID^{*}$, a timestamp $T^{*}$, and targeted public key $pk^{*}$. If $F(ID^{*})\neq 0\;\mathrm{mod}\;p$ or $K(m^{*})\neq 0\;\mathrm{mod}\;p$, C terminates; otherwise, C computes $h^{*}=H_{3}\left(m^{*},T^{*},ID^{*},pk^{*},\sigma_{2}^{*},\sigma_{3}^{*},param\right)$ and uses $\left(\theta_{1}^{*},\theta_{2}^{*},\theta_{3}^{*}\right)$ to output $g^{ab}$ as a solution to the CDH instance as follows:
  $$\begin{array}{cc} & \frac{\sigma_{1}^{*}}{g^{\theta_{1}^{*}\theta_{2}^{*}}{\left(\sigma_{2}^{*}\right)}^{J(ID^{*})}{\left(\sigma_{3}^{*}\right)}^{L\left(m^{*}\right)+h^{*}\theta_{3}^{*}}}\\ = & \frac{g_{2}^{a}g^{\theta_{1}^{*}\theta_{2}^{*}}{\left(U_{ID^{*}}\right)}^{r_{ID}^{*}}{\left({\left(pk_{3}^{*}\right)}^{h^{*}}V_{m^{*}}\right)}^{r_{m}^{*}}}{g^{\theta_{1}^{*}\theta_{2}^{*}}{\left(g^{r_{ID}^{*}}\right)}^{J(ID^{*})}{\left(g^{r_{m}^{*}}\right)}^{L\left(m^{*}\right)+h^{*}\theta_{3}^{*}}}\\ = & \frac{g_{2}^{a}{\left(g_{2}^{F(ID^{*})}g^{J(ID^{*})}\right)}^{r_{ID}^{*}}{\left({\left(g^{\theta_{3}^{*}}\right)}^{h^{*}}\left(g_{2}^{K(m^{*})}g^{L(m^{*})}\right)\right)}^{r_{m}^{*}}}{{\left(g^{J(ID^{*})}\right)}^{r_{ID}^{*}}{\left(g^{\theta_{3}^{*}}\right)}^{r_{m}^{*}h^{*}}{\left(g^{L(m^{*})}\right)}^{r_{m}^{*}}}\\ = & g_{2}^{a}g_{2}^{F\left(ID^{*}\right)r_{ID}^{*}}g_{2}^{K\left(m^{*}\right)r_{m}^{*}}\\ & (\mathrm{since}\;F\left(ID^{*}\right)=K\left(m^{*}\right)=0\;\mathrm{mod}\;p)\\ = & g_{2}^{a}\\ = & g^{ab}. \end{array}$$

Now, we analyze the probability that C can successfully solve the CDH problem. If the following conditions hold, C completes the above simulation without aborting.

(1) All partial private key queries on $\left(ID_{i},pk_{i}\right)$ have $F\left(ID_{i}\right)\neq 0\;\mathrm{mod}\;l_{u}$.
(2) All private key queries on $ID_{i}$ have $F\left(ID_{i}\right)\neq 0\;\mathrm{mod}\;l_{u}$.
(3) All signing queries on $\left(ID_{i},T,m\right)$ have $F\left(ID_{i}\right)\neq 0\;\mathrm{mod}\;l_{u}$ or $K\left(m\right)\neq 0\;\mathrm{mod}\;l_{m}$.
(4) In the forgery phase, $F(ID^{*})=0\;\mathrm{mod}\;p$ and $K(m^{*})=0\;\mathrm{mod}\;p$.

Here, we define four independent events $X_{i}$, $X^{*}$, $Y_{j}$, and $Y^{*}$ as follows.

- $X_{i}:\;F\left(ID_{i}\right)\neq 0\;\mathrm{mod}\;l_{u}$ for the *i*th query, where $1\leq i\leq q_{psk}+q_{sk}+q_{s}$.
- $X^{*}:\;F\left(ID^{*}\right)=0\;\mathrm{mod}\;p$.
- $Y_{j}:\;K\left(m_{j}\right)\neq 0\;\mathrm{mod}\;l_{m}$ for the *j*th query, where $1\leq j\leq q_{s}$.
- $Y^{*}:\;K\left(m^{*}\right)=0\;\mathrm{mod}\;p$.

Because the events ${⋂}_{i=1}^{q_{psk}+q_{sk}+q_{s}}X_{i}$, $X^{*}$, ${⋂}_{j=1}^{q_{s}}Y_{j}$, and $Y^{*}$ are independent, the probability that C does not terminate is

$$\begin{array}{cc} \mathrm{Pr}[\neg abort]\geq & \mathrm{Pr}[\overset{q_{psk}+q_{sk}+q_{s}}{\underset{i=1}{⋂}}X_{i}\cap X^{*}\cap \overset{q_{s}}{\underset{j=1}{⋂}}Y_{j}\cap Y^{*}]\\ = & \mathrm{Pr}\left[X^{*}\right]\cdot \mathrm{Pr}\left[\overset{q_{psk}+q_{sk}+q_{s}}{\underset{i=1}{⋂}}X_{i}|X^{*}\right]\cdot \mathrm{Pr}\left[Y^{*}\right]\cdot \mathrm{Pr}\left[\overset{q_{s}}{\underset{j=1}{⋂}}Y_{j}|Y^{*}\right]. \end{array}$$

From $l_{u}\left(n_{u}+1\right)<p$, $l_{m}\left(n_{m}+1\right)<p$, $0\leq k_{u}\leq n_{u}$, $0\leq k_{m}\leq n_{m}$, $x_{0},x_{1},\ldots ,x_{n_{u}}\in Z_{l_{u}}$ and $c_{0},c_{1},\ldots ,c_{n_{m}}\in Z_{l_{m}}$, we have $0\leq l_{u}k_{u}<p$, $0\leq l_{m}k_{m}<p$, $0\leq x_{0}+\sum_{i=1}^{n_{u}}x_{i}Q_{i}<p$ and $0\leq c_{0}+\sum_{j=1}^{n_{m}}c_{j}M_{j}<p$. Therefore, it is easy to derive $F\left(ID\right)=0\;\mathrm{mod}\;l_{u}$ and $K\left(m\right)=0\;\mathrm{mod}\;l_{m}$ from F(ID)=0modp and K(m)=0modp, respectively. Moreover, $F\left(ID\right)\neq 0\;\mathrm{mod}\;l_{u}$ implies that F(ID)≠0modp, and $K\left(m\right)\neq 0\;\mathrm{mod}\;l_{m}$ implies that K(m)≠0modp. Since $x_{0},x_{1},\ldots ,x_{n_{u}}$ and $c_{0},c_{1},\ldots ,c_{n_{m}}$ are randomly chosen, we obtain the probabilities of the events $X^{*}$ and $Y^{*}$ as follows:

$$\begin{array}{cc} \mathrm{Pr}[X^{*}]= & \mathrm{Pr}[F\left(ID^{*}\right)=0\;\mathrm{mod}\;p]\\ \geq & \mathrm{Pr}[F\left(ID^{*}\right)=0\;\mathrm{mod}\;p\cap F\left(ID^{*}\right)=0\;\mathrm{mod}\;l_{u}]\\ = & \mathrm{Pr}\left[F\left(ID^{*}\right)=0\;\mathrm{mod}\;l_{u}\right]\mathrm{Pr}\left[F\left(ID^{*}\right)=0\;\mathrm{mod}\;p|F\left(ID^{*}\right)=0\;\mathrm{mod}\;l_{u}\right]\\ = & \frac{1}{l_{u}}\frac{1}{n_{u}+1},\\ \mathrm{Pr}[Y^{*}]= & \mathrm{Pr}[K\left(m^{*}\right)=0\;\mathrm{mod}\;p]\\ \geq & \mathrm{Pr}[K\left(m^{*}\right)=0\;\mathrm{mod}\;p\cap K\left(m^{*}\right)=0\;\mathrm{mod}\;l_{m}]\\ = & \mathrm{Pr}\left[K\left(m^{*}\right)=0\;\mathrm{mod}\;l_{m}\right]\mathrm{Pr}\left[K\left(m^{*}\right)=0\;\mathrm{mod}\;p|K\left(m^{*}\right)=0\;\mathrm{mod}\;l_{m}\right]\\ = & \frac{1}{l_{m}}\frac{1}{n_{m}+1}. \end{array}$$

Furthermore, we have

$$\begin{array}{cc} \mathrm{Pr}[\overset{q_{psk}+q_{sk}+q_{s}}{\underset{i=1}{⋂}}X_{i}|X^{*}]= & 1-\mathrm{Pr}[\overset{q_{psk}+q_{sk}+q_{s}}{\underset{i=1}{⋃}}\neg X_{i}|X^{*}]\\ \geq & 1-\sum_{i=1}^{q_{psk}+q_{sk}+q_{s}}\mathrm{Pr}[\neg X_{i}|X^{*}]\\ = & 1-\frac{q_{psk}+q_{sk}+q_{s}}{l_{u}},\\ \mathrm{Pr}[\overset{q_{s}}{\underset{j=1}{⋂}}Y_{j}|Y^{*}]= & 1-\mathrm{Pr}[\overset{q_{s}}{\underset{j=1}{⋃}}\neg Y_{j}|Y^{*}]\\ \geq & 1-\sum_{j=1}^{q_{s}}\mathrm{Pr}[\neg Y_{j}|Y^{*}]=1-\frac{q_{s}}{l_{m}}. \end{array}$$

Since $l_{u}=2\left(q_{psk}+q_{sk}+q_{s}\right)$ and $l_{m}=2q_{s}$, we write

$$\begin{array}{cc} \mathrm{Pr}[\neg abort]\geq & \frac{1}{l_{u}}\cdot \frac{1}{n_{u}+1}\cdot \left(1-\frac{q_{psk}+q_{sk}+q_{s}}{l_{u}}\right)\frac{1}{l_{m}}\cdot \frac{1}{n_{m}+1}\cdot \left(1-\frac{q_{s}}{l_{m}}\right)\\ = & \frac{1}{16q_{s}\left(n_{u}+1\right)\left(n_{m}+1\right)\left(q_{psk}+q_{sk}+q_{s}\right)}. \end{array}$$

Therefore, if $A_{1}$ breaks the strong unforgeability of the proposed CLS scheme with advantage $\varepsilon_{1}$, then C has an advantage $\varepsilon_{1}^{{}^{\prime }}\geq$$\frac{\varepsilon_{1}}{16q_{s}\left(n_{u}+1\right)\left(n_{m}+1\right)\left(q_{psk}+q_{sk}+q_{s}\right)}$ to solve the given instance of the CDH problem. □

**Theorem** **2.** *In the standard model, the proposed CLS scheme is strongly unforgeable against MKGC attacks launched by the type II adversary $A_{2}$. Concretely, assuming that $A_{2}$ compromises the security of our CLS scheme with advantage $\varepsilon_{2}$ after making at most $q_{pk}$ public key queries, $q_{sk}$ private key queries, and $q_{s}$ signing queries, then an algorithm C can use $A_{2}$’s forgery to solve the CDH problem in $G_{1}$ with advantage $\varepsilon_{2}^{{}^{\prime }}$.*

**Proof.**  Supposing that a PPT adversary $A_{2}$ breaks the strong unforgeability of our CLS scheme in an adaptive chosen-message attack, we can construct an algorithm C that calls $A_{2}$ as a subroutine to violate the CDH assumption. Assuming that C is given a random instance $\left(g,A=g^{a},B=g^{b}\right)\in G_{1}^{3}$, to calculate $g^{ab}$, C simulates the challenger in Game 2 to answer all $A_{2}$’s queries.

- *Initialization*: For the given values $q_{sk}$, $q_{s}$, $n_{u}$, and $n_{m}$, C sets $l_{u}=2\left(q_{sk}+q_{s}\right)$ and $l_{m}=2q_{s}$ such that $l_{u}\left(n_{u}+1\right)<p$ and $l_{m}\left(n_{m}+1\right)<p$. C selects a random element $\theta^{*}\in Z_{p}^{*}$ and calculates $pk_{3}^{*}=g^{\theta^{*}}$. Then, C sets the targeted entity’s public key $pk^{*}=\left(pk_{1}^{*},pk_{2}^{*},pk_{3}^{*}\right)=\left(A=g^{a},B=g^{b},g^{\theta^{*}}\right)$ and sends parameters $\left(G_{1},G_{2},p,g,e\right)$ and $pk^{*}$ to $A_{2}$.
  Subsequently, $A_{2}$ performs the following steps to produce other system parameters and the master secret key.
  (1) Select two random integers $k_{u}$ and $k_{m}$, where $0\leq k_{u}\leq n_{u}$ and $0\leq k_{m}\leq n_{m}$.
  (2) Randomly select $x_{0},x_{1},\ldots ,x_{n_{u}}\in Z_{l_{u}}$, $c_{0},c_{1},\ldots ,$$c_{n_{m}}\in Z_{l_{m}}$ and $y_{0},y_{1}$, $\ldots ,y_{n_{u}}$, $d_{0},d_{1},\ldots$, $d_{n_{m}}\in Z_{p}$.
  (3) Select three collision-resistant hash functions $H_{1}:{\left\{0,1\right\}}^{*}\to {\left\{0,1\right\}}^{n_{u}}$, $H_{2}:{\left\{0,1\right\}}^{*}\to {\left\{0,1\right\}}^{n_{m}}$, and $H_{3}:{\left\{0,1\right\}}^{*}\to Z_{p}^{*}$.
  (4) Assign $u_{0}=B^{-l_{u}k_{u}+x_{0}}g^{y_{0}}$, $u_{i}=B^{x_{i}}g^{y_{i}}$ for $i=1,\ldots ,n_{u}$, $v_{0}=B^{-l_{m}k_{m}+c_{0}}g^{d_{0}}$, and $v_{j}=B^{c_{j}}g^{d_{j}}$ for $j=1,\ldots ,n_{m}$ and set $\vec{u}=\left(u_{1},\ldots ,u_{n_{u}}\right)$ and $\vec{v}=\left(v_{1},\ldots ,v_{n_{m}}\right)$.
  (5) Select two random values $\alpha ,\beta \in Z_{p}^{*}$ and compute $g_{1}=g^{\alpha }$, $g_{2}=g^{\beta }$ and $msk=g^{\alpha \beta }$.
  (6) Send parameters $\left(g_{1},g_{2},u_{0},v_{0},\vec{u},\vec{v},H_{1},H_{2},H_{3}\right)$ and the master secret key msk to C.
  Note that the secret value of the targeted entity is $usk^{*}=g^{ab}$, which is unknown to C, and the system parameters are $param=\{G_{1},G_{2},p,g,e,g_{1},g_{2},u_{0}$, $v_{0},\vec{u},\vec{v},H_{1},H_{2},H_{3}\}$.
  As the initialization phase in Theorem 1, we define the following four functions:
  $$\begin{array}{cc} F(ID) & =-l_{u}k_{u}+x_{0}+\sum_{i=1}^{n_{u}}x_{i}Q_{i},J\left(ID\right)=y_{0}+\sum_{i=1}^{n_{u}}y_{i}Q_{i},\\ K(m) & =-l_{m}k_{m}+c_{0}+\sum_{j=1}^{n_{m}}c_{j}M_{j},L\left(m\right)=d_{0}+\sum_{j=1}^{n_{m}}d_{j}M_{j}. \end{array}$$
  Furthermore, we have the following equations:
  $$\begin{array}{cc} U_{ID} & =u_{0}\prod_{i=1}^{n_{u}}u_{i}^{Q_{i}}=B^{F(ID)}g^{J(ID)},\\ V_{m} & =v_{0}\prod_{j=1}^{n_{m}}v_{j}^{M_{j}}=B^{K(m)}g^{L(m)}. \end{array}$$
- *Queries*: C maintains an initially empty list $L_{2}$ of tuples $\left\{\left(ID_{i},\theta_{i,1},\theta_{i,2},\theta_{i,3},pk_{i},sk_{i}\right)\right\}$ and builds the following oracles to answer the queries initiated by $A_{2}$.
  – *Public Key Query*$O_{pk}\left(ID_{i}\right)$: When $A_{2}$ issues such a query on an identity $ID_{i}$, C looks up the corresponding entry in list $L_{2}$ and sends $pk_{i}$ to $A_{2}$. Otherwise, if $L_{2}$ does not store this entry, C randomly selects $\theta_{i,1},\theta_{i,2},\theta_{i,3}\in Z_{p}^{*}$ and computes the public key $pk_{i}=\left(pk_{i,1},pk_{i,2},pk_{i,3}\right)=\left(A^{\theta_{i,1}},B^{\theta_{i,2}},g^{\theta_{i,3}}\right)=\left(g^{a\theta_{i,1}},g^{b\theta_{i,2}},g^{\theta_{i,3}}\right)$. Note that the secret value is $usk_{i}=g^{ab\theta_{i,1}\theta_{i,2}}$, but *a* and *b* are unknown to C. Then, C stores $\{(ID_{i}$, $\theta_{i,1},\theta_{i,2},\theta_{i,3},pk_{i})\}$ in $L_{2}$ and transmits $pk_{i}$ to $A_{2}$.
  – *Private Key Query*$O_{sk}\left(ID_{i}\right)$: Upon receipt of a query on an identity $ID_{i}$, C returns $sk_{i}$ to $A_{2}$ if $ID_{i}$ is found in $L_{2}$; otherwise, C makes a query $O_{pk}\left(ID_{i}\right)$ to obtain a public key $pk_{i}=\left(pk_{i,1},pk_{i,2},pk_{i,3}\right)$ and the triplet $\left(\theta_{i,1},\theta_{i,2},\theta_{i,3}\right)$ and then verifies whether $F\left(ID_{i}\right)=0\;\mathrm{mod}\;l_{u}$.
    (1) If $F\left(ID_{i}\right)=0\;\mathrm{mod}\;l_{u}$, C exits the simulation.
    (2) If $F\left(ID_{i}\right)\neq 0\;\mathrm{mod}\;l_{u}$, C selects $s_{i}\in Z_{p}^{*}$ and uses the master secret key $msk=g^{\alpha \beta }$ to compute
      $$\begin{array}{cc} sk_{i,1} & =A^{\frac{-J\left(ID_{i}\right)\theta_{i,1}\theta_{i,2}}{F(ID_{i})}}g^{\alpha \beta }{\left(U_{ID_{i}}\right)}^{s_{i}},\\ sk_{i,2} & =A^{\frac{-\theta_{i,1}\theta_{i,2}}{F(ID_{i})}}g^{s_{i}}, \end{array}$$
      where $U_{ID_{i}}=u_{0}\prod_{k=1}^{n_{u}}u_{k}^{Q_{i,k}}$ and ${\vec{Q}}_{i}=H_{1}(ID_{i}$, $pk_{i})=\left(Q_{i,1},\ldots ,Q_{i,n_{u}}\right)\in {\left\{0,1\right\}}^{n_{u}}$. Then, C stores the private key of the corresponding entry in $L_{2}$ and sends $sk_{i}=\left(sk_{i,1},sk_{i,2}\right)$ to $A_{2}$.
    The correctness of $sk_{i}$ simulated by C is
    $$\begin{array}{cc} sk_{i,1}= & A^{\frac{-J\left(ID_{i}\right)\theta_{i,1}\theta_{i,2}}{F(ID_{i})}}g^{\alpha \beta }{\left(U_{ID_{i}}\right)}^{s_{i}}\\ = & g^{\alpha \beta }g^{ab\theta_{i,1}\theta_{i,2}}{\left(g^{bF(ID_{i})}g^{J(ID_{i})}\right)}^{\frac{-a\theta_{i,1}\theta_{i,2}}{F(ID_{i})}}{\left(U_{ID_{i}}\right)}^{s_{i}}\\ = & g^{\alpha \beta }g^{ab\theta_{i,1}\theta_{i,2}}{\left(U_{ID_{i}}\right)}^{s_{i}-\frac{a\theta_{i,1}\theta_{i,2}}{F(ID_{i})}}\\ = & g^{\alpha \beta }g^{ab\theta_{i,1}\theta_{i,2}}{\left(U_{ID_{i}}\right)}^{{\tilde{s}}_{i}},\\ sk_{i,2}= & A^{\frac{-\theta_{i,1}\theta_{i,2}}{F(ID_{i})}}g^{s_{i}}=g^{s_{i}-\frac{a\theta_{i,1}\theta_{i,2}}{F(ID_{i})}}=g^{{\tilde{s}}_{i}}, \end{array}$$
    where ${\tilde{s}}_{i}=s_{i}-\frac{a\theta_{i,1}\theta_{i,2}}{F(ID_{i})}$. Hence, the above equations indicate that $sk_{i}=\left(sk_{i,1},sk_{i,2}\right)$ is a valid private key of identity $ID_{i}$.
  – *Signing Query*$O_{sign}\left(ID_{i},T,m\right)$: Upon receiving a message *m*, an identity $ID_{i}$, and a timestamp *T*, C issues a query $O_{pk}\left(ID_{i}\right)$ to obtain a public key $pk_{i}=\left(pk_{i,1},pk_{i,2},pk_{i,3}\right)$ and a triplet $\left(\theta_{i,1},\theta_{i,2},\theta_{i,3}\right)$. Then, C considers the following two cases:
    (1) If $F\left(ID_{i}\right)\neq 0\;\mathrm{mod}\;l_{u}$, C makes a query $O_{sk}\left(ID_{i}\right)$ to obtain a private key $sk_{i}$ and then runs the algorithm Sign to generate a signature $\sigma_{i}$ on *m*. Finally, C sends $\sigma_{i}$ to $A_{2}$.
    (2) If $F\left(ID_{i}\right)=0\;\mathrm{mod}\;l_{u}$, C computes K(m). If $K\left(m\right)=0\;\mathrm{mod}\;l_{m}$, C quits the simulation; otherwise, C randomly selects $r_{i},r_{m}\in Z_{p}^{*}$ and computes ${\vec{Q}}_{i}=H_{1}\left(ID_{i},pk_{i}\right)$, $U_{ID_{i}}$, $\vec{M}=H_{2}\left(m,T\right)$, and $V_{m}$. Furthermore, C computes
      $$\begin{array}{cc} \sigma_{i,2}= & g^{r_{i}},\\ \sigma_{i,3}= & A^{\frac{-\theta_{i,1}\theta_{i,2}}{K(m)}}g^{r_{m}},\\ h_{i}= & H_{3}\left(m,T,ID_{i},pk_{i},\sigma_{i,2},\sigma_{i,3},param\right),\\ \sigma_{i,1}= & g^{\alpha \beta }{\left(U_{ID_{i}}\right)}^{r_{i}}A^{\frac{\left(L\left(m\right)-h_{i}\theta_{i,3}\right)\theta_{i,1}\theta_{i,2}}{K(m)}}{\left({\left(pk_{i,3}\right)}^{h_{i}}V_{m}\right)}^{r_{m}}. \end{array}$$
      Finally, C sends $\sigma_{i}=\left(\sigma_{i,1},\sigma_{i,2},\sigma_{i,3}\right)$ to $A_{2}$.
  Let ${\tilde{r}}_{m}=r_{m}-\frac{a\theta_{i,1}\theta_{i,2}}{K(m)}$; then, we have
  $$\begin{array}{cc} \sigma_{i,1}= & g^{\alpha \beta }{\left(U_{ID_{i}}\right)}^{r_{i}}A^{\frac{\left(L\left(m\right)-h_{i}\theta_{i,3}\right)\theta_{i,1}\theta_{i,2}}{K(m)}}{\left({\left(pk_{i,3}\right)}^{h_{i}}V_{m}\right)}^{r_{m}}\\ = & g^{\alpha \beta }g^{ab\theta_{i,1}\theta_{i,2}}{\left(g^{bK(m)}g^{L(m)}g^{h_{i}\theta_{i,3}}\right)}^{\frac{-a\theta_{i,1}\theta_{i,2}}{K(m)}}{\left(U_{ID_{i}}\right)}^{r_{i}}{\left({\left(pk_{i,3}\right)}^{h_{i}}V_{m}\right)}^{r_{m}}\\ = & g^{\alpha \beta }g^{ab\theta_{i,1}\theta_{i,2}}{\left(\left(B^{K(m)}g^{L(m)}\right){\left(g^{\theta_{i,3}}\right)}^{h_{i}}\right)}^{\frac{-a\theta_{i,1}\theta_{i,2}}{K(m)}}{\left(U_{ID_{i}}\right)}^{r_{i}}{\left({\left(pk_{i,3}\right)}^{h_{i}}V_{m}\right)}^{r_{m}}\\ = & g^{\alpha \beta }g^{ab\theta_{i,1}\theta_{i,2}}{\left(U_{ID_{i}}\right)}^{r_{i}}{\left({\left(pk_{i,3}\right)}^{h_{i}}V_{m}\right)}^{r_{m}-\frac{a\theta_{i,1}\theta_{i,2}}{K(m)}}\\ = & g^{\alpha \beta }g^{ab\theta_{i,1}\theta_{i,2}}{\left(U_{ID_{i}}\right)}^{r_{i}}{\left({\left(pk_{i,3}\right)}^{h_{i}}V_{m}\right)}^{{\tilde{r}}_{m}},\\ \sigma_{i,2}= & g^{r_{i}},\\ \sigma_{i,3}= & A^{\frac{-\theta_{i,1}\theta_{i,2}}{K(m)}}g^{r_{m}}=g^{\frac{-a\theta_{i,1}\theta_{i,2}}{K(m)}}g^{r_{m}}=g^{r_{m}-\frac{a\theta_{i,1}\theta_{i,2}}{K(m)}}=g^{{\tilde{r}}_{m}}. \end{array}$$
  The simulated signature $\sigma_{i}=\left(\sigma_{i,1},\sigma_{i,2},\sigma_{i,3}\right)$ satisfies the following signature verification equation; thus, $\sigma_{i}$ is a valid signature on message *m*:
  $$\begin{array}{cc} e(\sigma_{i,1},g)= & e(g^{\alpha \beta }g^{ab\theta_{i,1}\theta_{i,2}}{\left(U_{ID_{i}}\right)}^{r_{i}}{\left({\left(pk_{i,3}\right)}^{h_{i}}V_{m}\right)}^{{\tilde{r}}_{m}},g)\\ = & e\left(g^{\alpha \beta },g\right)e\left(g^{ab\theta_{i,1}\theta_{i,2}},g\right)e\left({\left(U_{ID_{i}}\right)}^{r_{i}},g\right)e\left({\left({\left(pk_{i,3}\right)}^{h_{i}}V_{m}\right)}^{{\tilde{r}}_{m}},g\right)\\ = & e\left(g^{\alpha },g^{\beta }\right)e\left(g^{a\theta_{i,1}},g^{b\theta_{i,2}}\right)e\left(U_{ID_{i}},g^{r_{i}}\right)e\left({\left(pk_{i,3}\right)}^{h_{i}}V_{m},g^{{\tilde{r}}_{m}}\right)\\ = & e\left(g_{2},g_{1}\right)e\left(pk_{i,1},pk_{i,2}\right)e\left(U_{ID_{i}},\sigma_{i,2}\right)e\left({\left(pk_{i,3}\right)}^{h_{i}}V_{m},\sigma_{i,3}\right). \end{array}$$
- *Forgery*: $A_{2}$ eventually outputs a signature $\sigma^{*}=\left(\sigma_{1}^{*},\sigma_{2}^{*},\sigma_{3}^{*}\right)$ on a message $m^{*}$ corresponding to an identity $ID^{*}$, a timestamp $T^{*}$, and the targeted public key $pk^{*}$. If $F(ID^{*})\neq 0\;\mathrm{mod}\;p$ or $K(m^{*})\neq 0\;\mathrm{mod}\;p$, C terminates; otherwise, C calculates $h^{*}=H_{3}\left(m^{*},T^{*},ID^{*},pk^{*},\sigma_{2}^{*},\sigma_{3}^{*},param\right)$ and then uses $\theta^{*}$ and $g^{\alpha \beta }$ to output the CDH value $g^{ab}$ by calculating
  $$\begin{array}{cc} & \frac{\sigma_{1}^{*}}{g^{\alpha \beta }{\left(\sigma_{2}^{*}\right)}^{J(ID^{*})}{\left(\sigma_{3}^{*}\right)}^{L\left(m^{*}\right)+h^{*}\theta^{*}}}\\ = & \frac{g^{\alpha \beta }g^{ab}{\left(U_{ID^{*}}\right)}^{r_{ID}^{*}}{\left({\left(pk_{3}^{*}\right)}^{h^{*}}V_{m^{*}}\right)}^{r_{m}^{*}}}{g^{\alpha \beta }{\left(g^{r_{ID}^{*}}\right)}^{J(ID^{*})}{\left(g^{r_{m}^{*}}\right)}^{L\left(m^{*}\right)+h^{*}\theta^{*}}}\\ = & \frac{g^{ab}{\left(B^{F(ID^{*})}g^{J(ID^{*})}\right)}^{r_{ID}^{*}}{\left({\left(pk_{3}^{*}\right)}^{h^{*}}\left(B^{K(m^{*})}g^{L(m^{*})}\right)\right)}^{r_{m}^{*}}}{{\left(g^{J(ID^{*})}\right)}^{r_{ID}^{*}}{\left(g^{\theta^{*}}\right)}^{r_{m}^{*}h^{*}}{{\left(g^{r_{m}^{*}}\right)}^{r_{m}^{*}}}^{L(m^{*})}}\\ = & g^{ab}B^{F\left(ID^{*}\right)r_{ID}^{*}}B^{K\left(m^{*}\right)r_{m}^{*}}\\ & (\mathrm{since}\;F\left(ID^{*}\right)=K\left(m^{*}\right)=0\;\mathrm{mod}\;p)\\ = & g^{ab}. \end{array}$$

Here, we discuss the probability of C outputting a correct solution for the CDH instance. C completes the above simulation if all of the following events occur:

- $F\left(ID_{i}\right)\neq 0\;\mathrm{mod}\;l_{u}$ during private key queries.
- $F\left(ID_{i}\right)\neq 0\;\mathrm{mod}\;l_{u}$ or $K\left(m\right)\neq 0\;\mathrm{mod}\;l_{m}$ during signing queries.
- $F(ID^{*})=0\;\mathrm{mod}\;p$ and $K(m^{*})=0\;\mathrm{mod}\;p$ in the forgery phase.

The probability of C completing the simulation is analogous to that in Theorem 1. We define four independent events, $X_{i}$, $X^{*}$, $Y_{j}$, and $Y^{*}$, as follows:

- $X_{i}:\;F\left(ID_{i}\right)\neq 0\;\mathrm{mod}\;l_{u}$ for $1\leq i\leq q_{sk}+q_{s}$.
- $X^{*}:\;F\left(ID^{*}\right)=0\;\mathrm{mod}\;p$.
- $Y_{j}:\;K\left(m_{j}\right)\neq 0\;\mathrm{mod}\;l_{m}$ for $1\leq j\leq q_{s}$.
- $Y^{*}:\;K\left(m^{*}\right)=0\;\mathrm{mod}\;p$.

Similar to the probability analysis in Theorem 1, we give the probability of C not aborting as

$$\begin{array}{cc} \mathrm{Pr}[\neg abort]= & \mathrm{Pr}[\overset{q_{sk}+q_{s}}{\underset{i=1}{⋂}}X_{i}\cap X^{*}\cap \overset{q_{s}}{\underset{j=1}{⋂}}Y_{j}\cap Y^{*}]\\ = & \mathrm{Pr}\left[X^{*}\right]\mathrm{Pr}\left[\overset{q_{sk}+q_{s}}{\underset{i=1}{⋂}}X_{i}|X^{*}\right]\mathrm{Pr}\left[Y^{*}\right]\mathrm{Pr}\left[\overset{q_{s}}{\underset{j=1}{⋂}}Y_{j}|Y^{*}\right]\\ = & \frac{1}{l_{u}\left(n_{u}+1\right)}\left(1-\frac{q_{sk}+q_{s}}{l_{u}}\right)\frac{1}{l_{m}\left(n_{m}+1\right)}\left(1-\frac{q_{s}}{l_{m}}\right)\\ = & \frac{1}{16q_{s}\left(q_{sk}+q_{s}\right)\left(n_{u}+1\right)\left(n_{m}+1\right)}. \end{array}$$

Hence, C can solve the given instance of the CDH problem with advantage $\varepsilon_{2}^{{}^{\prime }}\geq \frac{\varepsilon_{2}}{16q_{s}\left(q_{sk}+q_{s}\right)\left(n_{u}+1\right)\left(n_{m}+1\right)}$. □

We obtain Theorem 3 by combining Theorems 1 and 2, as follows.

**Theorem** **3.** *In the standard model, our CLS scheme is strongly unforgeable against adaptive chosen-message attacks corresponding to type I and II adversaries under the CDH and CRHF assumptions.*

**Theorem** **4.** *Our CLS scheme is resistant to replay attacks.*

**Proof.**  In replay attacks, the adversary generally initiates two types of attacks [31,32]. One is to directly replay the intercepted message and the corresponding signature, and the other is to modify the timestamp in the signature of the intercepted message and to create a new signature for the message.

In the first type of attack, it is assumed that the adversary replays an intercepted combination of message *m*, timestamp *T*, and signature σ generated by an IoT device. Upon receiving this combination {m,T,σ}, the data centre compares the timestamp *T* in the combination with the current timestamp $T^{{}^{\prime }}$. If the value of $T^{{}^{\prime }}-T$ exceeds the threshold δ, the data centre can determine that *m* in this combination is a replayed message and can discard *m*. Therefore, the first type of attack has no effect on our CLS scheme. □

Since our CLS scheme satisfies strong unforgeability, the attacker cannot generate a legal signature for any message. Therefore, in the second type of attack, the attacker can only use the existing combination {m,T,σ} to initiate the attack. In the proposed scheme, the timestamp *T* is bound to the message *m*, i.e., $\vec{M}=H_{2}\left(m,T\right)$. Additionally, the timestamp *T* is embedded in the parameter *h* in the form of $h=H_{3}\left(m,T,ID,pk_{ID},\sigma_{2},\sigma_{3},param\right)$ and is also embedded in the signature σ of the message *m* in the form of ${\left(pk_{3}\right)}^{r_{m}h}$. If the attacker wants to replace *T* in the signature σ with a new timestamp $T^{*}$, the attacker needs to calculate $h^{*}=H_{3}\left(m,T^{*},ID,pk_{ID},\sigma_{2},\sigma_{3},param\right)$ and ${\left(pk_{3}\right)}^{r_{m}h^{*}}$. Although an attacker can calculate $h^{*}$ and ${\left(pk_{3}\right)}^{h^{*}}$, the difficulty in calculating $r_{m}$ from ${\left(pk_{3}\right)}^{r_{m}h}$ is equivalent to solving the DL problem. However, if the attacker does not know $r_{m}$, then they cannot calculate the correct value ${\left(pk_{3}\right)}^{r_{m}h^{*}}$. In addition, $T^{*}$ must satisfy the conditions $H_{2}\left(m,T\right)=H_{2}\left(m,T^{*}\right)$ and $H_{1}\left(m,T\right)=H_{1}\left(m,T^{*}\right)$, which is equivalent to finding a collision of the hash functions $H_{2}$ and $H_{3}$. Since the DL problem is unsolvable in reality and the functions $H_{2}$ and $H_{3}$ are CRHF, the second type of attack does not compromise the security of our CLS scheme. In summary, the proposed CLS scheme can efficiently withstand replay attacks.

## 6. Application in IoT Environments and Performance Analysis

### 6.1. System Model

**Figure 2:**
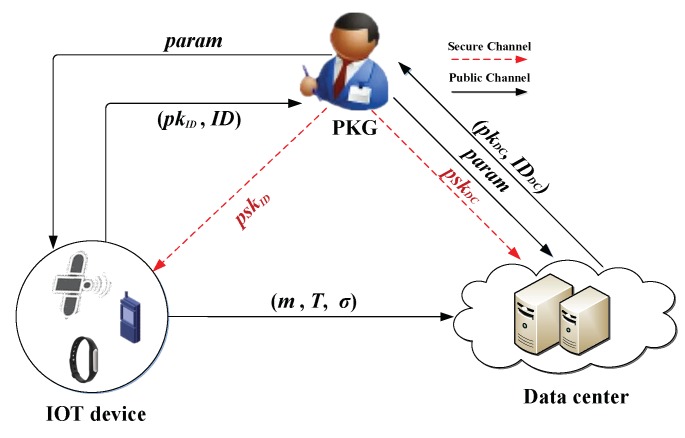
System model of the proposed certificateless signature (CLS) scheme for IoT.

In a CLS scheme for IoT environments, it is very important that data are not modified and that the source of the data is authentic during data transmission. Therefore, we mainly focus on the integrity and authenticity of IoT data in our system while simultaneously reducing the bandwidth, computational cost, and storage overhead for IoT devices. Figure 2 shows our CLS system model for IoT environments, which consists of three entities: PKG, data centre, and IoT device.

- **PKG:** This entity is primarily responsible for producing system parameters and computing partial private keys for the data centre and each IoT device. The PKG sends system parameters to all of the entities through a public channel and transmits an individual partial private key to each entity via a secure channel.
- **Data centre:** This entity has a strong computing power and storage space; thus, it can check the integrity and authenticity of the data by verifying the signature sent by each IoT device and can store the authentic data for other users to use. Initially, the data centre submits its identity information to the PKG to apply for the corresponding partial private key; it then saves the system parameters and partial private key sent by the PKG.
- **IoT device:** This entity equipped with sensors has limited computational and memory resources and limited battery capacity. During the registration of the IoT device, the PKG generates a unique partial private key based on the physical address of each IoT device. After the IoT device is embedded with system parameters and its private key, it signs messages collected from the physical world and sends the corresponding signatures along with messages to the data centre.

### 6.2. Performance Analysis

In this subsection, we analyze the performance of the proposed CLS scheme. Compared with other cryptographic operations, bilinear pairing and exponentiation are the most time-consuming operations [22,28]; hence, our efficiency analysis mainly emphasizes the computational costs of these two operations. Table 1 and Table 2 compare the performance of our CLS scheme and other related CLS schemes [21,22,23,27,30] without random oracles in terms of private key size, signature length, computational cost, and security. In Table 1, the *KeySize* and *SigSize* columns list the sizes of the private key and signature, respectively. The *Sign* and *Verify* columns present the computational costs of the algorithms Sign and Verify, respectively. Let *P* and *E* represent the execution times of a bilinear pairing and an exponentiation, respectively. Let $n_{u}$ represent the length of an identity, and let $|G_{1}|$ and |p| represent the lengths of an element in $G_{1}$ and $Z_{p}$, respectively. In Table 2, the columns *Type I*, *Type II*, and *Replay attacks* show whether the CLS scheme can resist PKR attacks, MKGC attacks, and replay attacks, respectively. The *SUF* column denotes whether the CLS scheme satisfies a strong unforgeability in the standard model. It should be noted that the key length affects the storage capacity of the IoT device and the data center and that the signature length affects the communication capabilities of the IoT device and the storage capacity of the data center. In addition, the overhead of signature generation and signature verification affect the computing power of the IoT device and the data center, respectively.

From Table 1 and Table 2, the length of the private key in our CLS scheme is $2|G_{1}|$, which is the shortest among the six CLS schemes. The size of the signature in the proposed CLS scheme is $3|G_{1}|$, which is equivalent to that of the schemes presented in References [22,23,27] but smaller than that of other schemes [21,30]. In the signing phase, our CLS scheme requires three exponentiations, as does Yuan et al.’s scheme [23], but is superior to other schemes [21,22,27,30]. In the verification phase, the computational cost of the proposed CLS scheme is E+5P, which is lower than that of the five other CLS schemes. Moreover, the efficiency of the verification process in our CLS scheme can be improved by a pre-calculation. Note that the verification equation for signature legitimacy is as follows:

$$\begin{array}{c} e\left(\sigma_{1},g\right)=e\left(g_{2},g_{1}\right)\cdot e\left(pk_{ID,1},pk_{ID,2}\right)\cdot e\left(U_{ID},\sigma_{2}\right)\cdot e\left({\left(pk_{ID,3}\right)}^{h}V_{m},\sigma_{3}\right). \end{array}$$

Here, $e(g_{2},g_{1})$ and $e(pk_{ID,1},pk_{ID,2})$ can be pre-computed; thus, the time cost of verification in our CLS scheme can be reduced to one exponentiation and 3 bilinear pairings. Furthermore, only our CLS scheme can resist PKR attacks, MKGC attacks, and replay attacks while satisfying a strong unforgeability.

**Figure 3:**
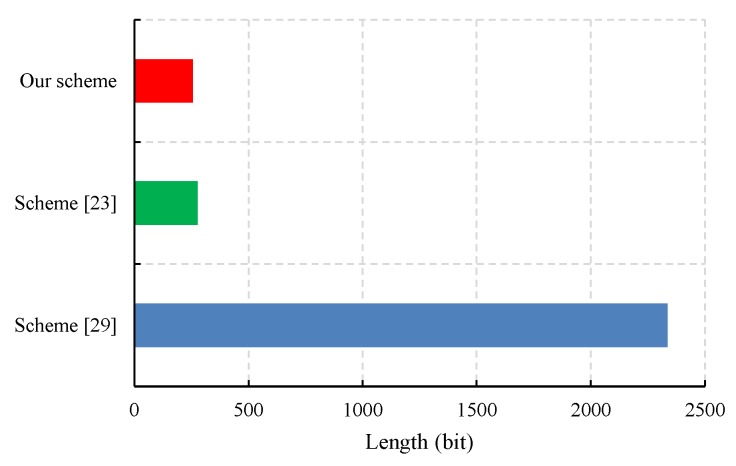
A comparison of the private key size.

**Figure 4:**
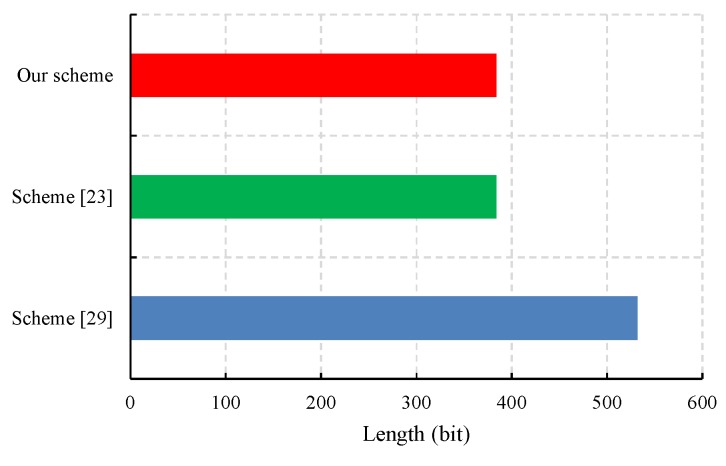
A comparison of the communication cost.

**Figure 5:**
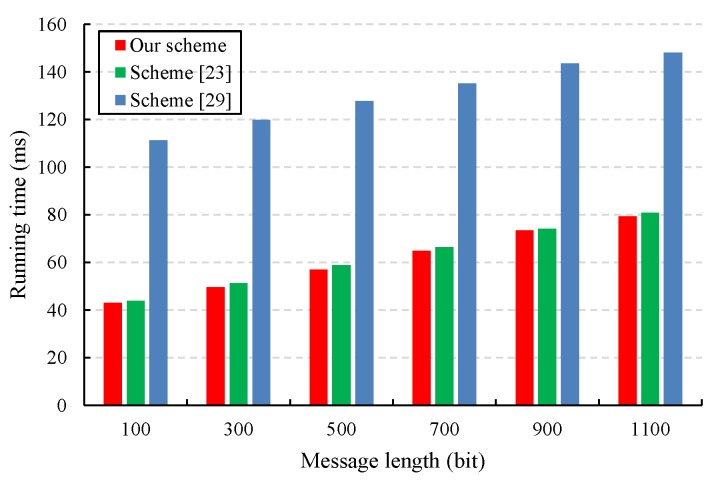
A comparison of the signature generation cost.

**Figure 6:**
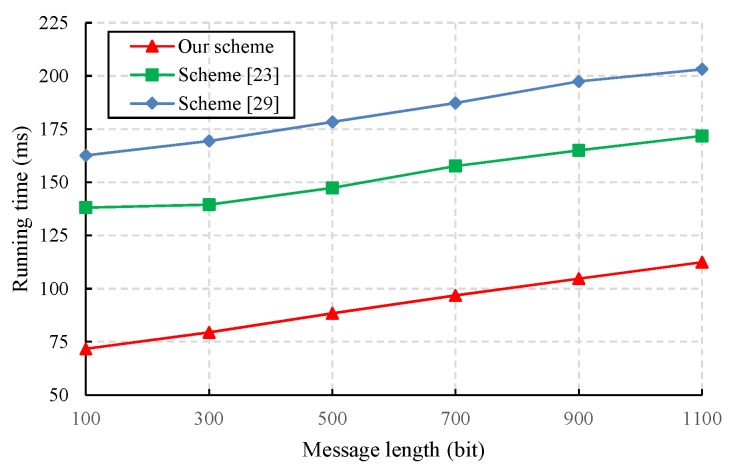
A comparison of the signature verification cost.

We also evaluated the performance of the proposed CLS scheme via experiments conducted with the PBC-0.47-VC cryptographic library [50]. The simulation program was run on a laptop equipped with a basic configuration of a 2.50 GHz CPU, 8 GB RAM, and the 64-bit Windows 10 operating system. To obtain faster pairing computation, we selected the Type A curve in the PBC library, which is a super-singular curve $y^{2}=x^{3}+x$ built with the 512-bit order of the base field. The results of the experiment are presented in Figure 3, Figure 4, Figure 5 and Figure 6.

The IoT device must secretly store its private key; therefore, the size of the private key is important for an IoT device with a limited storage capacity. As shown in Figure 3, the size of the private key in our CLS scheme is 256 bits, which is 92.8% of that in Yuan et al.’s CLS scheme [23]. However, the size of the private key increases linearly with the length of the entity’s identity in Yang et al.’s CLS scheme [30]. For example, if the length $n_{u}$ of the entity’s identity is 100 bits, then the private key size in our CLS scheme is approximately 11% of that in Yang et al.’s CLS scheme [30]. In other words, our CLS scheme has a higher performance in private key length.

Since IoT devices possess limited battery power and communication bandwidth, one of the goals of our CLS scheme is to reduce the communication overhead of IoT devices. The most critical factor affecting communication cost is signature size. Figure 4 shows that the signature size of our CLS scheme and that of Yuan et al. [23] is 384 bits, while the signature size of Yang et al.’s CLS scheme [30] is 532 bits. Hence, the proposed CLS scheme has a lower communication overhead.

Due to the characteristics of IoT devices, such as limited computing and processing power, the computational overhead of generating signatures for IoT devices should be as small as possible. Figure 5 shows that the cost of signature generation in our CLS scheme is almost the same as that in Yuan et al.’s CLS scheme [23] but less than that in Yang et al.’s CLS scheme [30].

The data centre has a strong computation and storage capability to verify the validity of signatures sent by IoT devices. Figure 6 shows that the proposed CLS scheme greatly reduces the computational overhead of signature verification and that its performance is superior to that of the other two schemes [23,30].

**Figure 7:**
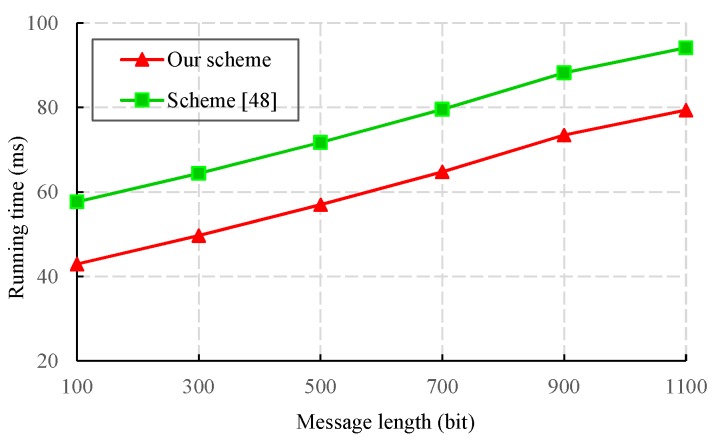
A comparison of the signature generation cost between CLS-based and SDVPRS-based authentication schemes.

**Figure 8:**
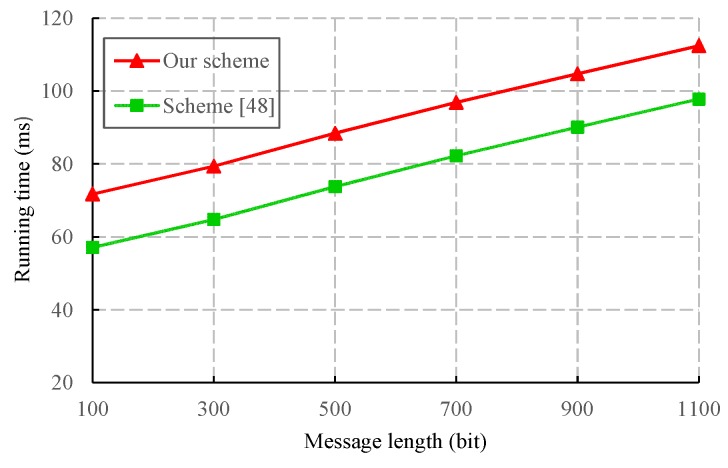
A comparison of the signature verification cost between CLS-based and SDVPRS-based authentication schemes.

A scheme in the random oracle model usually has a higher computational performance, but its security depends on the ideal random oracle. Both our scheme and Yang et al.’s [48] scheme are provable in the standard model, and their security only depends on the difficulty of the associated mathematical problems. Therefore, these two schemes have higher security than other schemes [4,40,41,42,44,46,47]. Our scheme and Yang et al.’s scheme [48] use CLS and SDVPRS respectively to guarantee the integrity and authenticity of data in IoT. We compare the signature generation and verification overhead of two schemes, and the corresponding results are shown in Figure 7 and Figure 8.

From Figure 7, we can see that the computational cost of signature generation in our scheme is lower than that in the scheme of Reference [48]. This is because the signature generation in the scheme of Reference [48] requires an additional bilinear pairing operation. Figure 8 shows that the time consumption of signature verification in the scheme of Reference [48] is lower than ours, but the scheme in Reference [48] does not satisfy the properties of a strong unforgeability and replay attack resistance. As a result, our scheme has a higher security.

In summary, the results of all the above experimental analyses are consistent with those of the theoretical analysis in Table 1. Therefore, we conclude that our CLS scheme is applicable to IoT environments.

## 7. Conclusions

The IoT is profoundly changing production activities, social management, and public services, but ensuring the integrity and authenticity of data is an important issue for IoT. To solve this problem, a new CLS scheme for IoT environments is presented in this paper. In addition to protecting data integrity and data authenticity, our CLS scheme also reduces the computational and communication costs for IoT devices. The proposed CLS scheme is proven to be strongly unforgeable against adaptive chosen-message attacks under the CDH and CRHF assumptions in the standard model. Additionally, our CLS scheme can withstand replay attacks. Furthermore, the performance comparisons demonstrate that our CLS scheme outperforms the previous CLS schemes without random oracles. The Internet of Vehicles is considered to be one of the most potential areas in IoT and has wide application prospects in the field of intelligent transportation. Compared with ordinary sensors, the vehicle terminal equipment has a more stable power supply and higher computing power and storage space. Hence, our CLS scheme is suitable for the Internet of Vehicles. 

## Figures and Tables

**Table 1:** A comparison of the CLS scheme performance.

Scheme	KeySize	SigSize	Sign	Verify
Yu et al. [21]	$\left|p|+2\right|G_{1}|$	$4|G_{1}|$	7E	E+5P
Yuan et al. [23]	$\left|p|+2\right|G_{1}|$	$3|G_{1}|$	3E	E+6P
Pang et al. [27]	$\left|p|+2\right|G_{1}|$	$3|G_{1}|$	7E	4E+5P
Huang et al. [22]	$3|G_{1}|$	$3|G_{1}|$	5E	3E+6P
Yang et al. [30]	$\left(4+n_{u}\right)\left|p|+2\right|G_{1}|$	$\left|p|+4\right|G_{1}|$	10E	3E+7P
Our scheme	$2|G_{1}|$	$3|G_{1}|$	3E	E+3P

**Table 2:** A comparison of the security attributes.

Scheme	Type I	Type II	SUF	Replay Attacks
Yu et al. [21]	No	No	No	No
Yuan et al. [23]	Yes	Yes	No	No
Pang et al. [27]	Yes	Yes	No	No
Huang et al. [22]	Yes	No	No	No
Yang et al. [30]	Yes	Yes	Yes	No
Our scheme	Yes	Yes	Yes	Yes
